# Systems analysis of avascular necrosis of femoral head using integrative data analysis and literature mining delineates pathways associated with disease

**DOI:** 10.1038/s41598-020-75197-0

**Published:** 2020-10-22

**Authors:** Ashwin Ashok Naik, Aswath Narayanan, Prakash Khanchandani, Divya Sridharan, Piruthivi Sukumar, Sai Krishna Srimadh Bhagavatam, Polani B. Seshagiri, Venketesh Sivaramakrishnan

**Affiliations:** 1 Disease Biology Lab, Department of Biosciences, Sri Sathya Sai Institute of Higher Learning, Prasanthinilayam, Andhra Pradesh 515 134 India; 2 Department of Orthopedics, Sri Sathya Sai Institute of Higher Medical Sciences, Prasanthigram, Andhra Pradesh 515 134 India; 3 Molecular Reproduction and Developmental Genetics, Indian Institute of Science, Bangalore, Bangalore, India; 4 Leeds Institute of Cardiovascular and Metabolic Medicine, School of Medicine, University of Leeds, Leeds, LS2 9JT UK

**Keywords:** Computational biology and bioinformatics, Systems biology, Diseases, Pathogenesis

## Abstract

Avascular necrosis of femoral head (AVNFH) is a debilitating disease, which affects the middle aged population. Though the disease is managed using bisphosphonate, it eventually leads to total hip replacement due to collapse of femoral head. Studies regarding the association of single nucleotide polymorphisms with AVNFH, transcriptomics, proteomics, metabolomics, biophysical, ultrastructural and histopathology have been carried out. Functional validation of SNPs was carried out using literature. An integrated systems analysis using the available datasets might help to gain further insights into the disease process. We have carried out an analysis of transcriptomic data from GEO-database, SNPs associated with AVNFH, proteomic and metabolomic data collected from literature. Based on deficiency of vitamins in AVNFH, an enzyme-cofactor network was generated. The datasets are analyzed using ClueGO and the genes are binned into pathways. Metabolomic datasets are analyzed using MetaboAnalyst. Centrality analysis using CytoNCA on the data sets showed cystathionine beta synthase and methylmalonyl-CoA-mutase to be common to 3 out of 4 datasets. Further, the genes common to at least two data sets were analyzed using DisGeNET, which showed their involvement with various diseases, most of which were risk factors associated with AVNFH. Our analysis shows elevated homocysteine, hypoxia, coagulation, Osteoclast differentiation and endochondral ossification as the major pathways associated with disease which correlated with histopathology, IHC, MRI, Micro-Raman spectroscopy etc. The analysis shows AVNFH to be a multi-systemic disease and provides molecular signatures that are characteristic to the disease process.

## Introduction

Avascular Necrosis of Femoral Head (AVNFH) is a debilitating disease, usually affecting younger individuals. Clinically AVNFH is associated with reduction in vascular supply to the subchondral bone of the femoral head which leads to necrosis and collapse of articular surface and eventual degeneration of hip joint^1–3^. AVNFH has been categorized basically into idiopathic and secondary varieties. The secondary causes of AVNFH include haemoglobinopathies, storage disorders, SLE, decompression sickness etc.^4,5^ alcohol, Steroid, trauma, coagulopathies^6,7^ Numerous other factors like genetic factors, environmental factors, physiological process like pregnancy, lifestyle involving smoking, drug abuse, diseases like haemochromatosis have also been implicated as risk factors in AVNFH^8–10^.

Biophysical characterization of bone from AVNFH patients and piglet model of disease shows reduced mineral to matrix ratio and increased carbonate to phosphate ratio indicative of extensive remodelling due to osteoclast activity^1,11^. Histopathology shows increased cement lines and lacunae while electron microscopy shows micro-cracks, dead bone and woven bone substitution^1^. Immuno histochemistry of AVNFH bone shows increased osteocalcein^1^, iNOS^12^, RANKL^13^, BMP-2 and HIF-1 staining^14,15^. AVNFH is also associated with coagulation disorders and coagulopathies possibly play an important role in the pathophysiology of the disease. Consistent with this, increased microparticles have been observed in the blood of AVNFH patients which might clog capillaries and contribute to avascularity^14–17^. High amounts of Von Willebrand Factor which is associated with AVNFH will also lead to thrombosis^18^. Coagulation factor disorders have been correlated with AVNFH^19^. Endochondral ossification, where cartilage is replaced by bone was also shown to be inhibited in AVNFH^20^. These studies point to a complex association of hypoxia, thrombosis, reduced osteoblast activity and increased osteoclast activity in the pathogenesis of AVNFH.

More so, AVNFH has now been considered as a Multi systemic disease rather than as a disease involving just the femoral head^4^. The genetic factors include mutations in Col2A2, MTHFR, NOS, PAI-1, PON-1, alpha-2-Microglobulin, SREBP-2, VEGFC, ADH-2, and sometimes associated with Increased Lipoprotein A and protein C deficiency^21^. However, MTHFR mutations do not correlate with the disease in Korean and Japanese populations^22^, pointing to a possible population specific differences.

Omic techniques like transcriptomic, proteomic or metabolomics analysis of serum or tissues from AVNFH patients or model systems have been carried out^23–26^. The transcriptomic and proteomic analysis have largely reiterated on the signalling pathways that are deregulated in AVNFH^24,26^. These pathways are largely binned into osteoclastogenesis, osteoblasts and their function etc. Microarray studies have been carried out using rat models of steroid induced AVNFH and cartilage from diseased area of human AVNFH patients^27^. The results show deregulation of key pathways involved in the pathogenesis of AVNFH. Microarray analysis of whole transcriptome of AVNFH articular cartilage was performed at different time points from the induction of ischemic injury and compared to control^24^. The study showed genes involved in ischemic injury and angiogenesis were significantly upregulated at 2 weeks post AVNFH onset and those belonging to the inflammatory pathway four weeks post onset of the disease. Proteomic studies have also been carried out on AVNFH patient bone samples^28^. These studies points to deregulation of multiple signalling and metabolic pathways which are implicated in cartilage and bone biology. Targeted metabolomic analysis shows elevated levels of metabolites belonging to Methionine–Homocysteine pathway, Polyamine and urea pathway concomitant with low levels of B_12_, B_6_ and Betaine in AVNFH patients^1^. Metabolomic analysis of bone tissue in Chinese population showed changes in amino-acid and fatty acid metabolism^25^. Serum metabolomic analysis of rabbit model of steroid induced AVNFH showed differentially regulated phospholipids^29^. These studies show perturbations in different signalling and metabolic pathways in AVNFH.

However, a concerted effort to analyse the disease from a systems perspective by integrating the data sets from omic-studies as well as data from those reported in literature has largely been lacking. In the present study an integrative analysis of AVNFH associated SNPs, transcriptomic data sets of bone and cartilage, proteomic data sets and metabolomic data have been carried out. The data is analysed using cytoscape and binned into different pathways using its plugin ClueGO. The SNP data was validated by literature search for experimentally verified effects or phenotypes. The deficiency of vitamins/cofactors like B_6_, B_12_ and Folate and its implications for enzyme activity concomitant with deregulation of metabolic pathways has been analysed. The implications of the deficiency of these vitamins has been analysed in the light of transcriptomic and proteomic data obtained from cartilage or bone from patients and animal models. Further, the SNP data, transcriptomic data and proteomic data have been integrated with Vitamin-cofactor enzyme data. The role of SNPs, transcriptomic, proteomic and metabolomic data and its role in osteoblast, osteoclast and cartilage biology, their function as well as its implications for AVNFH has been discussed. The data analysis shows deregulation of multiple pathways consistent with the multi-systemic nature of AVNFH. In addition, the genes from integrative analysis common to at least two data sets were analysed using DisGeNET. The results show the involvement of these genes with various diseases. These diseases are risk factors associated with AVNFH. Further, we validated the role of elevated homocysteine on MSC differentiation to osteoblast and mineralization. We also show that RANKL is elevated in the AVNFH patients. Using literature mining we show elevated homocysteine, vitamin B_6_, B_12_, glutathione, N-acetylcysteine, CBS and MUT Knock out and knock down modulate RANKL induced osteoclastogenesis. Our analysis provides a theoretical basis to understand the disease and raises further questions which could shed light on the disease process. This could in turn provide potential biomarkers and newer therapeutic targets in the disease.

## Results

### SNPs associated with AVNFH and their functional implication

**Figure 1:**
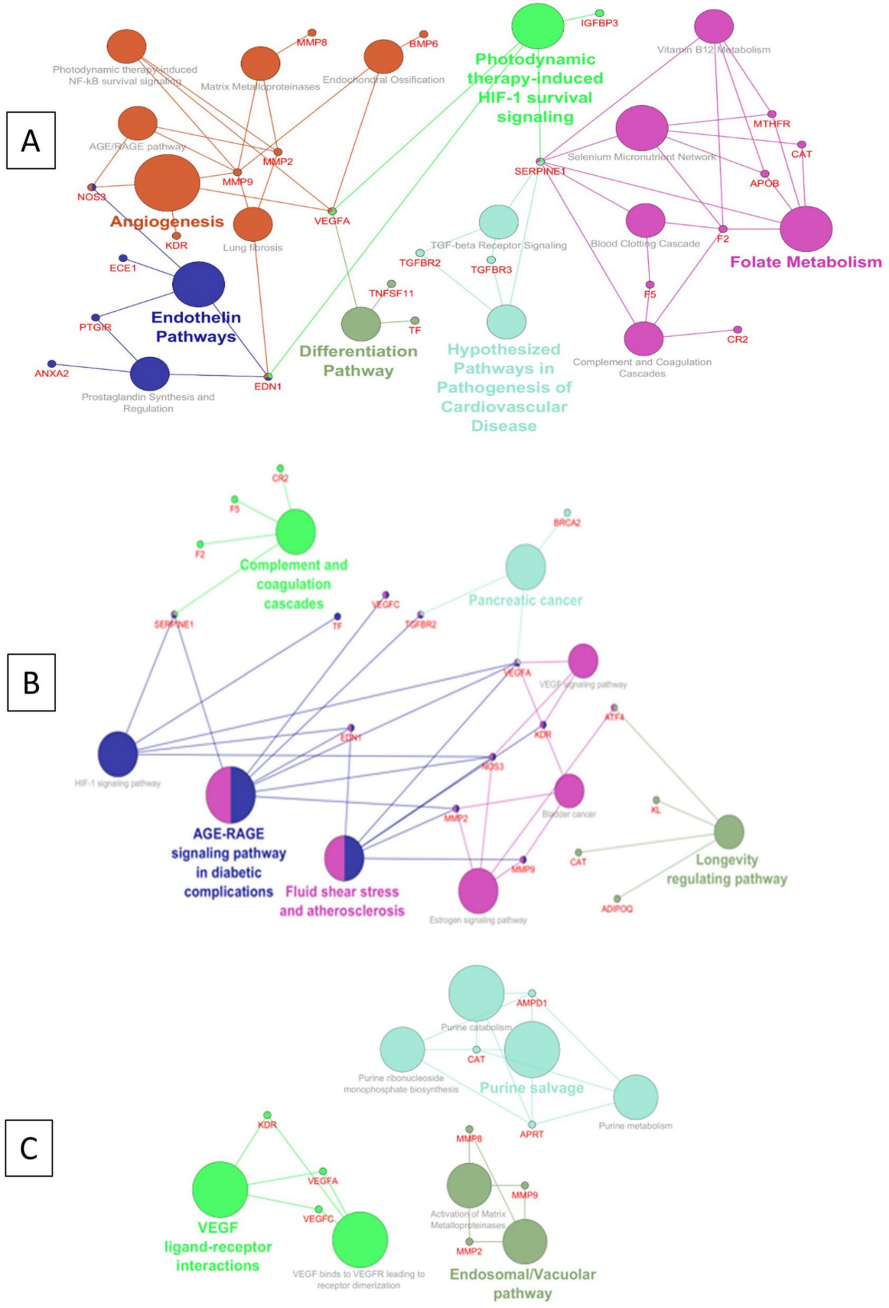

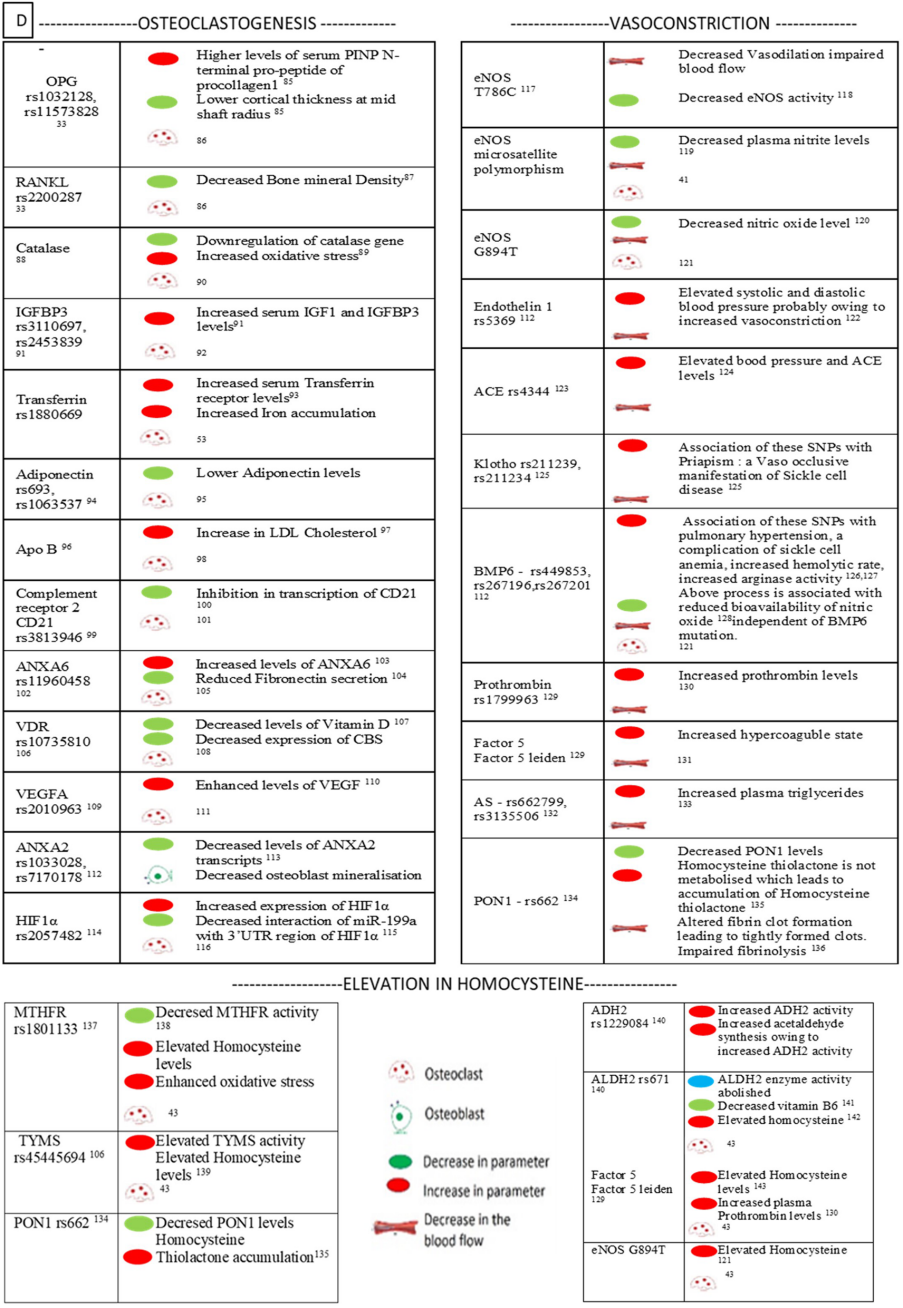
Pathway annotation analysis and functional implications of AVNFH SNP genes. (**A**) WikiPathways showing involvement of Endochondral Ossification, Angiogenesis, Differentiation pathway, Vitamin B_12_, Folate metabolism, Blood clotting, Complement and coagulation cascade. (**B**) KEGG pathways showing fluid shear stress and atherosclerosis. (**C**) Reactome pathways showing VEGF ligand–receptor interactions. (**D**) Functional implications of AVNFH SNPs ascertained from literature (References are provided under supplementary references S7).

The SNPs were curated from literature. A total of 57 Genes harbouring SNPs associated with AVNFH were curated. To evaluate the importance of SNPs in the disease an interaction network was generated using cytoscape and pathway annotation analysis was carried out using the Plugin ClueGO. The analysis using WikiPathways shows that the genes with SNPs associated with AVNFH are binned into Vitamin B_12_ metabolism, Folate metabolism, TGFβ signalling, matrix metallo proteases, Clotting and coagulation, HIF-1 pathway, angiogenesis, Endochondral ossification etc. The Reactome pathway binned genes into vacuolar pathway, matrix metallo proteinases, VEGF and purine metabolism. The KEGG pathway binned genes into Coagulation cascade, Fluid shear stress and atherosclerosis, HIF-1 pathway, VEGF signalling and AGE/RAGE pathway (Fig. 1A–C, Supplementary Data S1). Further, to evaluate the functional implication of SNPs in genes associated with AVNFH, we manually curated the experimentally verified SNPs with demonstrated impaired function from literature. Of the 57 curated genes harbouring SNPs associated with AVNFH, 26 genes harbouring 38 SNPs were found to have experimentally validated functional consequences. These SNPs were found to be associated with elevated levels of homocysteine, osteoclastogenesis and vasoconstriction. The summary of the genes involved in these processes are provided in Fig. 1D.

### Transcriptomic analysis of AVNFH cartilage shows deregulation in multiple pathways

**Figure 2:**
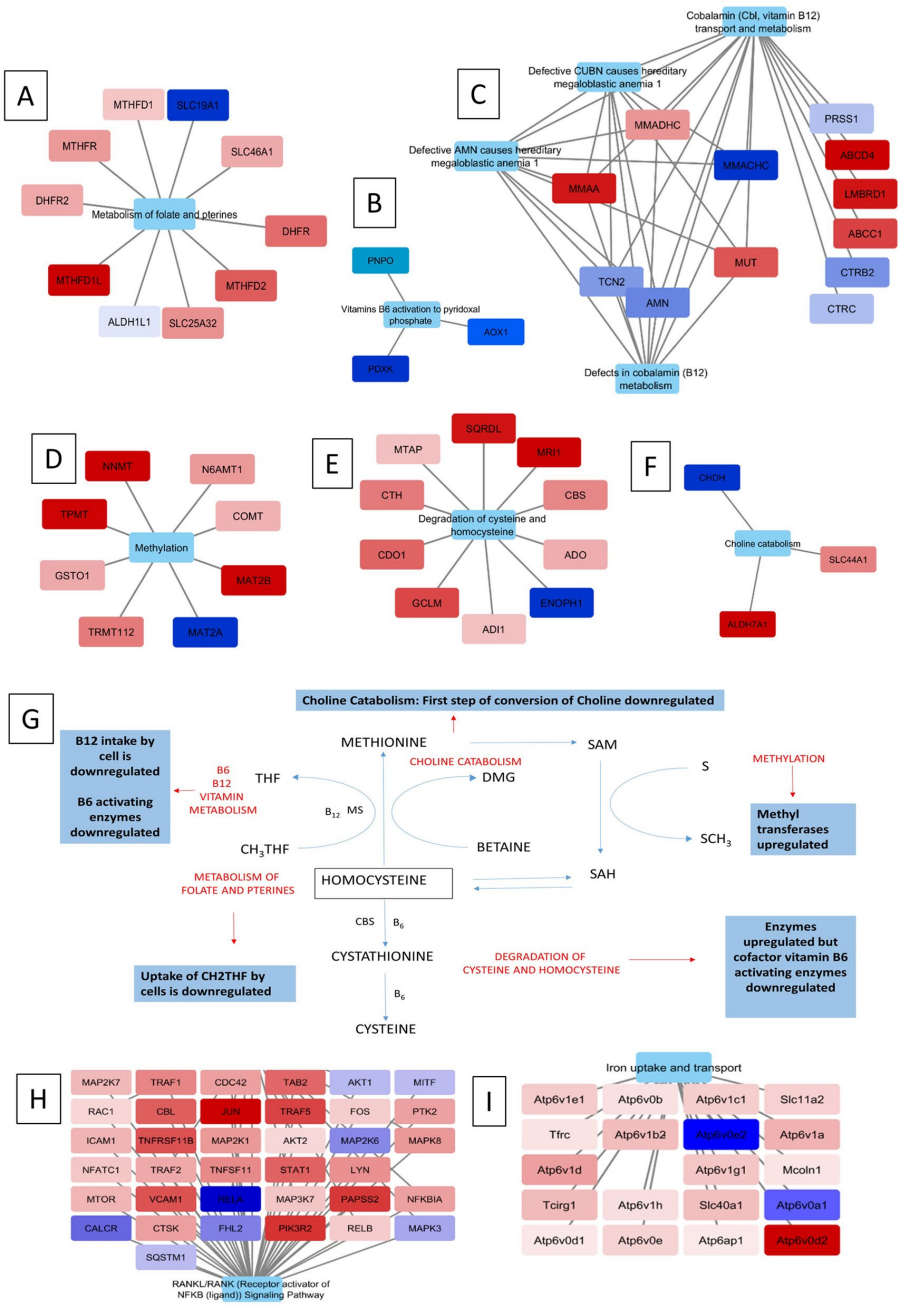
Pathway annotation analysis of AVNFH patient cartilage transcriptomics and RANKL induced osteoclastogenesis transcriptomics. (**A**–**C**) Metabolism of water soluble vitamins and cofactors pathway sub networks showing differentially regulated genes belonging to (**A**) folate metabolism, (**B**) vitamin B_6_, (**C**) vitamin B_12_. (**D**) Phase II conjugation of compounds sub network showing methylation pathway. (**E**,**F**) Metabolism of amino acids and derivatives sub networks showing (**E**) degradation of cysteine and Homocysteine pathway and (**F**) choline catabolism pathway (red—upregulated genes; blue—downregulated genes). (**G**) Summary of findings showing defective vitamin metabolism could lead to Homocysteine accumulation. (**H**) RANKL/RANK signaling in AVNFH transcriptomics. (**I**) Iron uptake and transport pathway in RANKL induced osteoclastogenesis transcriptomics.

Transcriptomic analysis was carried out on differential gene expression data sets from AVNFH patient cartilage compared to that of controls (GSE74089). The genes with an adjusted P. value of ≤ 0.05 were selected for analysis using cytoscape. The genes are subsequently binned into pathways using the plugin ClueGO. The results of analysis using KEGG shows deregulation of signalling and metabolic pathways having significant term P. value like alanine aspartate and glutamate metabolism, glycolysis/gluconeogenesis, methionine and cysteine metabolism, B_6_, B_12_ and Folate (Water soluble vitamin), alcoholism, estrogen pathway etc. (Supplementary Data S2). Analysis using WikiPathways shows deregulation of pathways like Tryptophan and histidine catabolism, RANKL/RANK signalling Pathway, endochondral ossification, Hematopoietic Stem Cell Differentiation, adipogenesis, Folate-Alcohol and Cancer Pathway, Iron metabolism, Blood Clotting Cascade and Folate Metabolism (Supplementary Data S2). Analysis using Reactome pathways shows deregulation of pathways like Metabolism of water soluble vitamins and cofactors, Metabolism of amino acids and derivatives, Phase II conjugation. Regulation of HIF by oxygen, signalling by VEGF, Collagen degradation, etc. (Supplementary Data S2). Further, subnetwork analysis for Reactome pathways was carried out using Reactome pathways browser (Supplementary Figures S7.1 and S7.3). Subnetwork analysis of water soluble vitamins and cofactors shows enrichment of Metabolism of folate and pterines, vitamin B_6_ activation to pyridoxal phosphate, Cobalamin (B_12_) transport and metabolism as well as (Fig. 2A–C). Phase II conjugation of compounds showing methylation pathway (Fig. 2D). Subnetwork analysis of metabolism of amino acids and derivatives shows enrichment of degradation of cysteine and homocysteine pathways, choline catabolism (Fig. 2E,F). The changes in gene expression in various deregulated pathways has been analysed for relevance for various metabolic pathways. The deregulation of B_12_, B_6_, Folate and Choline metabolism had major implications for the degradation of cysteine and homocysteine pathway as well as methylation reaction. The results are summarised in Fig. 2G.

### Proteomic analysis of AVNFH Bone shows deregulation in multiple pathways

**Figure 3:**
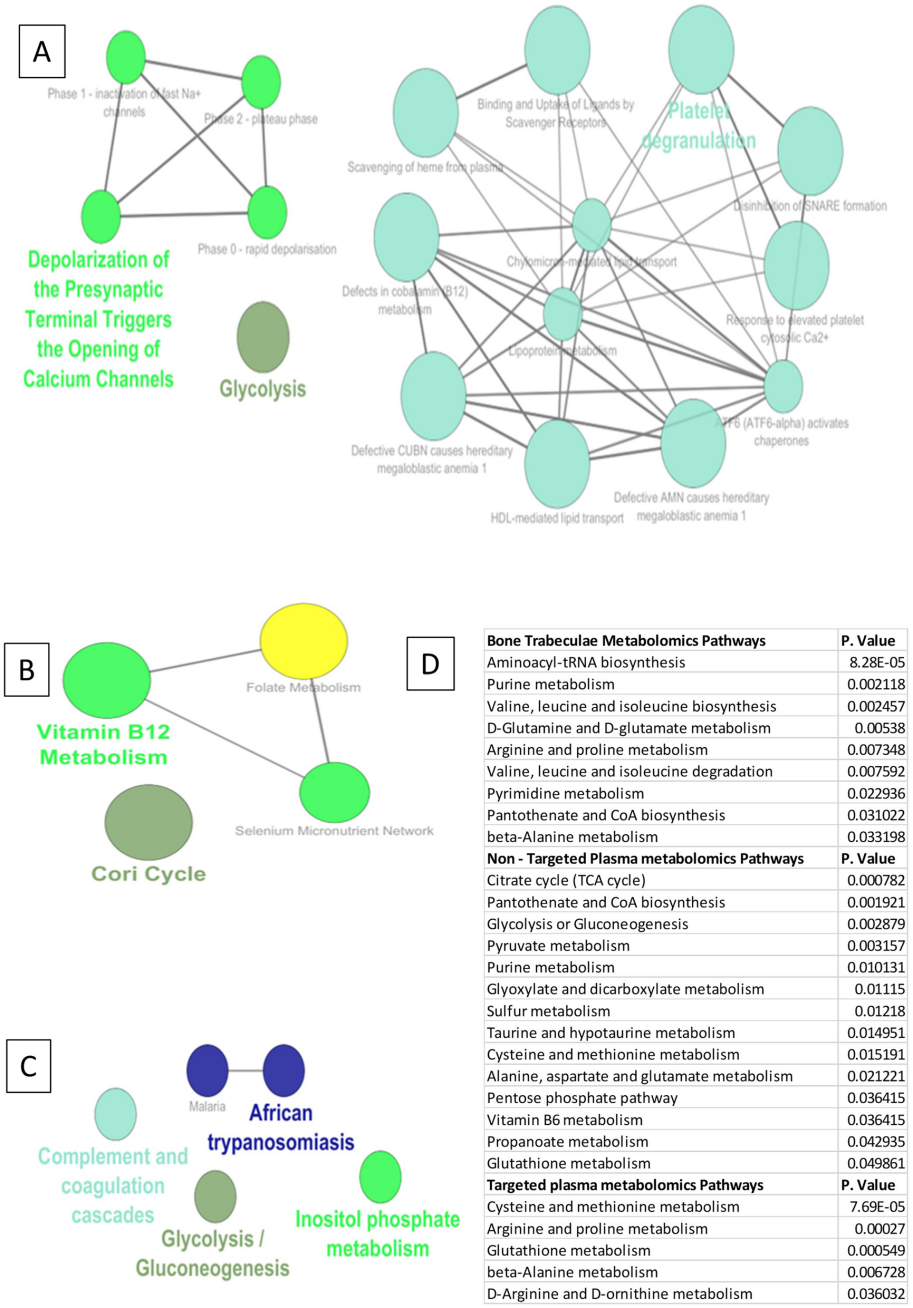
Pathway annotation analysis of AVNFH patient bone tissue proteomics. (**A**) Reactome pathways showing defects in Cobalamin metabolism, Scavenging heme from plasma, Megaloblastic anemia, Platelet degranulation pathways. (**B**) WikiPathways showing vitamin B_12_ and folate metabolism. (**C**) KEGG pathways showing complement and coagulation cascade pathway. (**D**) Pathway annotation analysis of AVNFH metabolomics showing significant pathways of patient targeted plasma metabolomics, significant pathways of patient non targeted plasma metabolomics and significant pathways of patient bone trabeculae metabolomics.

Proteomic functional analysis was carried out using published dataset for bone tissue affected with AVNFH compared to healthy controls^26^. The dataset showed 128 significantly upregulated proteins and 52 significantly downregulated proteins. The proteins with significant changes in the levels of expression were used for pathway annotation analysis using ClueGO a plugin of Cytoscape. Analysis using Reactome pathways binned the proteins into pathways involving—defects in cobalamin (B_12_) metabolism, Platelet degranulation, scavenging of Heme from plasma, Defective AMN causes hereditary megaloblastic anemia, and Glycolysis pathways (Fig. 3A and Supplementary Data S3). Analysis using WikiPathways binned the proteins into pathways involving Cori cycle, Selenium micronutrient network, Vitamin B_12_ metabolism, Folate metabolism etc. (Fig. 3B and Supplementary Data S3). Analysis using KEGG pathways binned the proteins into pathways involving Complement and coagulation cascades, Glycolysis/ Gluconeogenesis, inositol phosphate metabolism etc. (Fig. 3C and Supplementary Data S3).

### Metabolomic studies on AVNFH shows changes in critical metabolites involved in bone biology

From our previous study using plasma from AVNFH patient compared to age and gender matched control we have shown elevated levels of metabolites belonging to methionine-homocysteine pathway concomitant with a reduction in B_6_, B_12_ and betaine^1^. Metabolites belonging to polyamine and urea pathway was also found to be deregulated^1^. Metabolomics of plasma of AVNFH showed 48 differentially regulated metabolites^23^ while that of bone trabeculae showed 53 differentially regulated metabolites^25^. Pathway annotation analysis of these metabolites belonging to individual cohorts were carried out using MetaboAnalyst^30^. The published metabolomic data from bone trabeculae shows changes in aminoacid metabolism, purine and pyrimidine metabolism, Steroid, thymine and quinone metabolism^25^. The results of MetaboAnalyst are provided in Fig. 3D. Different metabolites influence bone biology by modulating osteoblast and osteoclast differentiation and their function.

### Low levels of vitamin B_6_, B_12_ and folate or defects in expression of genes that maintain normal physiological levels of vitamins affect activity of different enzymes belonging to multiple metabolic pathways

**Figure 4:**
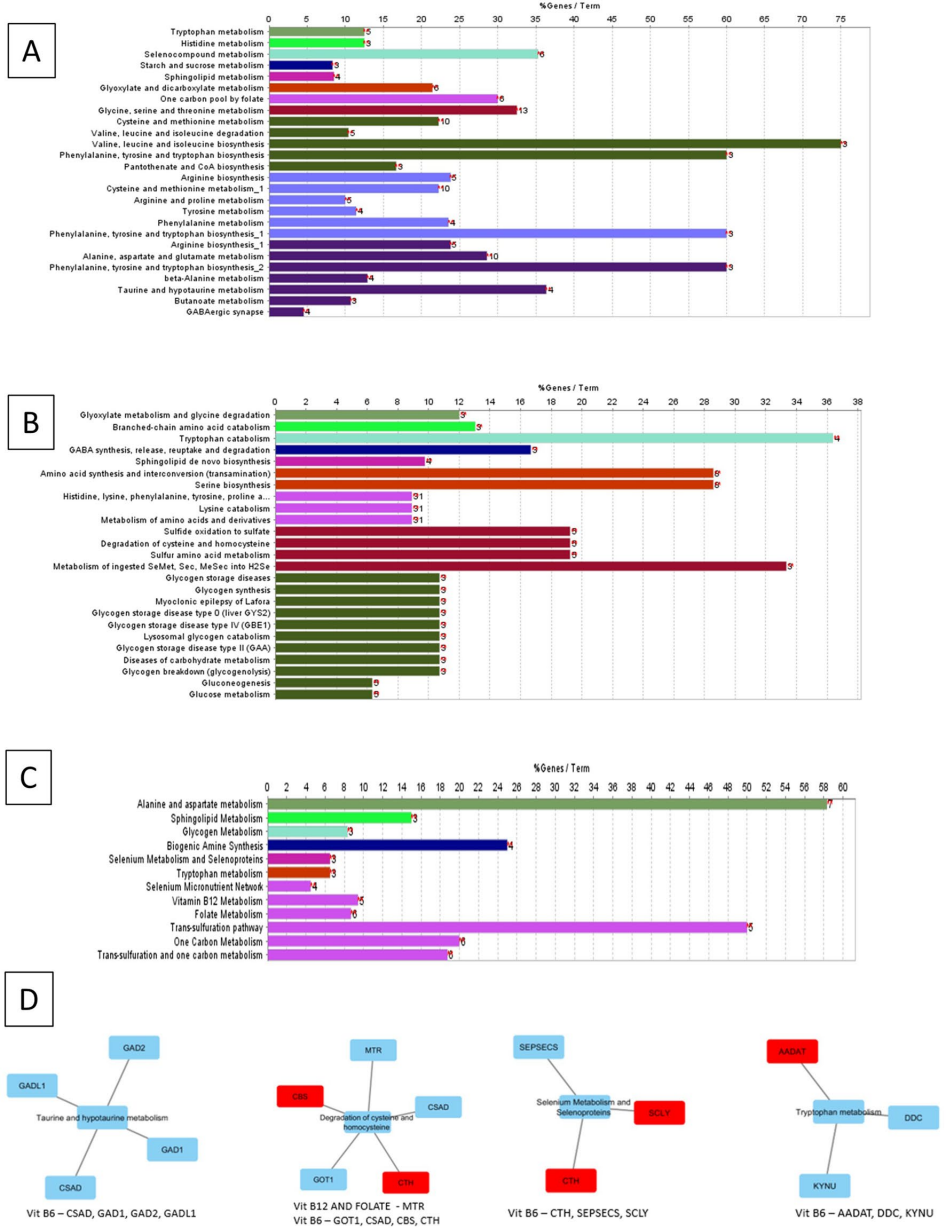
Vitamin B_6_, B_12_ and folate cofactor dependent proteins network and pathway annotation analysis. (**A**) KEGG pathways showing one carbon pool by folate, Cysteine and Methionine metabolism, Taurine and Hypotaurine metabolism. (**B**) Reactome pathways showing degradation of cysteine and homocysteine pathway. (**C**) WikiPathways showing Vitamin B_12_, folate, Transulfuration and one carbon metabolism pathway. (**D**) Overlay of gene expression values onto cofactor—protein pathways showing upregulated genes (Red—upregulated).

Data analysis from published Transcriptomic, proteomic and metabolomic data was used to arrive at the cofactors that are deregulated in AVNFH. The data from published literature involving cofactors and their respective enzymes^31^ were used to understand the role of deficiency in cofactors in AVNFH (B_6_, B_12_, Folate). An interaction network of the cofactors and the enzymes were generated and pathway annotation analysis was carried out using ClueGO. The results of this analysis for B_6_, B_12_, Folate shows enrichment in amino acid metabolism, Fatty acid and steroid metabolism, taurine and hypotaurine metabolism, one carbon metabolism, cysteine and homocysteine metabolism, selenocysteine metabolism, glycogen and glucose metabolism (Fig. 4A–C and Supplementary Data S4). Subsequently, to evaluate if the enzymes which uses cofactors like B_6_, B_12_, folate are deregulated in AVNFH, we imported the gene expression or protein expression data sets into the enzyme-cofactor interaction network. Many of the enzymes belonging to the enzyme-cofactor network were found to be upregulated in AVNFH and the genes were subsequently binned into pathways. The analysis also showed that though the enzymes were upregulated in AVNFH the dysfunction might mainly stem from deficiency of the cofactors (Fig. 4D).

### RANKL induced osteoclastogenesis shows changes in the expression levels of genes belonging to choline metabolism and iron metabolism

Homocysteine induces RANKL expression and secretion in osteoblasts and synovial fibroblasts^32^. In addition, SNPs in RANKL and OPG are found to be associated with AVNFH^33^. Transcriptomic analysis also shows enrichment of genes belonging to RANK-RANKL signaling (Fig. 2H). Hence, we analyzed transcriptomics, studying the role of RANKL in osteoclastogenesis. The evidence for increased osteoclastogenesis is also reiterated by Micro-Raman spectroscopy, ultrastructural studies, histopathology and IHC from previous studies^1^. Analysis of RANKL induced osteoclastogenesis shows enrichment of genes belonging to Choline metabolism, Heme degradation, single carbon metabolism and Iron metabolism (Fig. 2I and Supplementary Data S5). SNPs in genes implicated in Iron metabolism is associated with AVNFH. More so, many diseases like sickle cell anemia, beta-thalassemia and hemochromatosis are associated with AVNFH. All these diseases show iron overload and increased osteoclastogenesis or presence of osteoclast markers in the serum^9,10^.

### Integrative analysis of SNPs associated with AVNFH, Transcriptomic and Proteomic data as well as Cofactor-Enzyme data sets shows common pathways critical for progression of AVNFH which is also reflected in histopathology, IHC, Micro-Raman spectroscopy, CT and MRI of AVNFH patients or animal models

**Figure 5:**
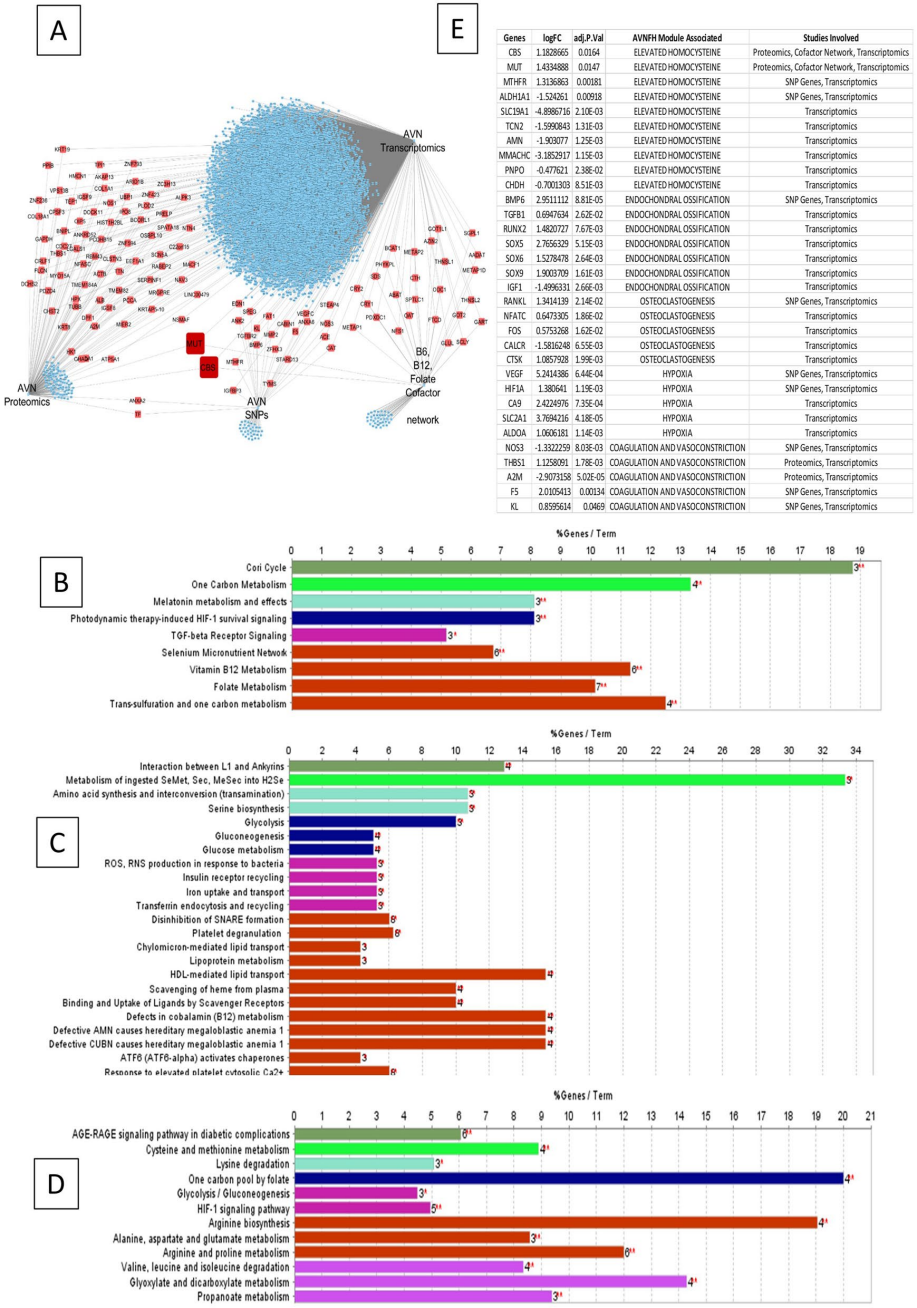
Integration of AVNFH omic analyses: (**A**) Integrated network of AVNFH transcriptomics, Proteomics, SNP genes and cofactor—protein network. Red nodes are hub genes, among which CBS and MUT showed highest network centrality scores. (**B**) WikiPathways annotation analysis of hub genes showing Vitamin B_12_, folate metabolism, transulfuration and one carbon metabolism. (**C**) Reactome pathways showing Iron uptake, transferrin endocytosis, scavenging heme from plasma, megaloblastic anemia pathways. (**D**) KEGG pathways showing HIF signaling, cysteine and methionine metabolism, one carbon pool by folate pathways. (**E**) Gene expression values of genes belonging to different modules like elevated homocysteine, hypoxia, osteoclastogenesis, endochondral ossification as well as coagulation and vasoconstriction from transcriptomics analysis.

The integrated network was obtained by merging individual networks of AVNFH SNPs, Proteomic, transcriptomic, and cofactor—protein networks using cytoscape. The network centrality analysis gave the degree, closeness and betweenness scores of individual nodes. 123 nodes had degree 2 (the genes were common in at least two data sets used). Two of the nodes, CBS and MUT had degree 3. Defective MUT and CBS due to Vitamin B_12_ and B_6_ deficiency respectively leads to accumulation of Methyl malonic acid and Homocysteine which stimulate osteoclastogenesis^34,35^ (Fig. 5A and Supplementary Data S6). These 125 hub nodes were used for ClueGO functional analyses. The integrative pathway annotation analysis with WikiPathways shows changes in Cori cycle, one carbon metabolism, TGFβ signaling, trans-sulfuration pathway, vitamin metabolism etc., (Fig. 5B and Supplementary Data S6). Reactome shows changes in amino acid metabolism, glucose metabolism, platelet degranulation, Iron and heme metabolism, single carbon metabolism, megaloblastic anemia, platelet degranulation etc., (Fig. 5C and Supplementary Data S6). Pathway annotation analysis using KEGG shows perturbations in amino acid metabolism including cysteine and methionine metabolism, HIF signaling, AGE-RAGE signaling pathway etc., (Fig. 5D and Supplementary Data S6). Further, we also looked at the expression levels of genes belonging to different modules like elevated homocysteine, hypoxia, osteoclastogenesis, endochondral ossification as well as coagulation and vasoconstriction. The expression levels of these genes as well as their statistical significance are provided from transcriptomic analysis (Fig. 5E). These genes could be of potential use for any future investigation to address the role of different modules in AVNFH.

In addition, the data from AVNFH patients and animal model systems for MRI, CT, Histopathology, IHC and Micro-Raman Spectroscopy of bone was correlated with molecular signatures obtained from –Omic analysis. Micro-Raman spectroscopy of human AVNFH bone and piglet AVNFH model showed reduced mineral matrix ratio and increased carbonate to phosphate ratio indicative of increased osteoclastogenesis and decreased osteoblast function (Supplementary Table S7.5 and references there in). The molecular signatures of increased osteoclastogenesis and homocysteine mediated inhibition of mineralization by osteoblast correlate with these results (Supplementary Table S7.5 and references there in). Histopathology of AVNFH bone reveals increased cement lines, empty lacunae and marrow calcification, which correlate with the molecular features like osteoclastogenesis, endochondral ossification and elevated homocysteine (Supplementary Table S7.5 and references there in). Consistent with this IHC studies have revealed changes in RANK, RANKL, Osteocalcein, BMP2 etc. which are indicative of bone remodeling, increased osteoclast and osteoblast activity (Supplementary Table S7.5 and references there in). MRI-BOLD of AVNFH patients show significant changes in oxygen content of arterial and venous blood. MRI data also shows changes in velocity of blood flow (Supplementary Table S7.5 and references there in). Histopathology shows coagulation and vasoconstriction in AVNFH patients (Supplementary Table S7.5 and references there in). The molecular signature of increased coagulation, atherosclerosis, platelet degranulation, elevated homocysteine, Von Willebrand factor and microparticles correlate with the findings of MRI and Histopathology (Supplementary Table S7.5 and references there in). The coagulation process also leads to hypoxia (Supplementary Table S7.5 and references there in) which corroborate with increased HIF-1α and VEGF staining in AVNFH bone using IHC (Supplementary Table S7.5 and references there in). The SNP and transcriptomic data also bins genes into hypoxia signaling pathway in AVNFH.

**Figure 6:**
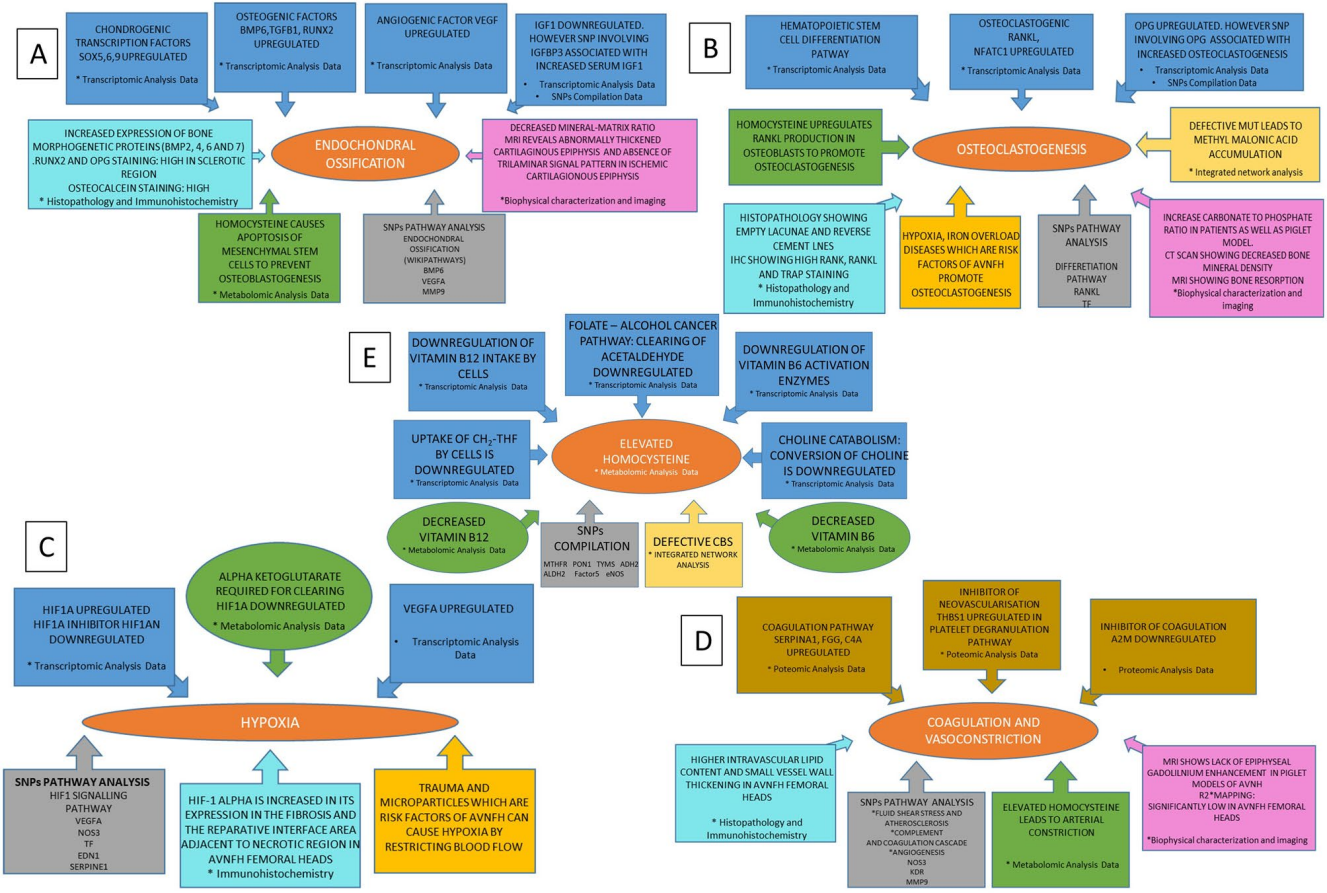
Modules of salient features of AVNFH addressed in our study by analysing various omic data and their integration with published results of histopathology, IHC, MRI, CT and Micro-Raman Spectroscopy. (**A**) Endochondral ossification, (**B**) osteoclastogenesis, (**C**) hypoxia, (**D**) coagulation and vasoconstriction, (**E**) elevation of homocysteine.

**Figure 7:**
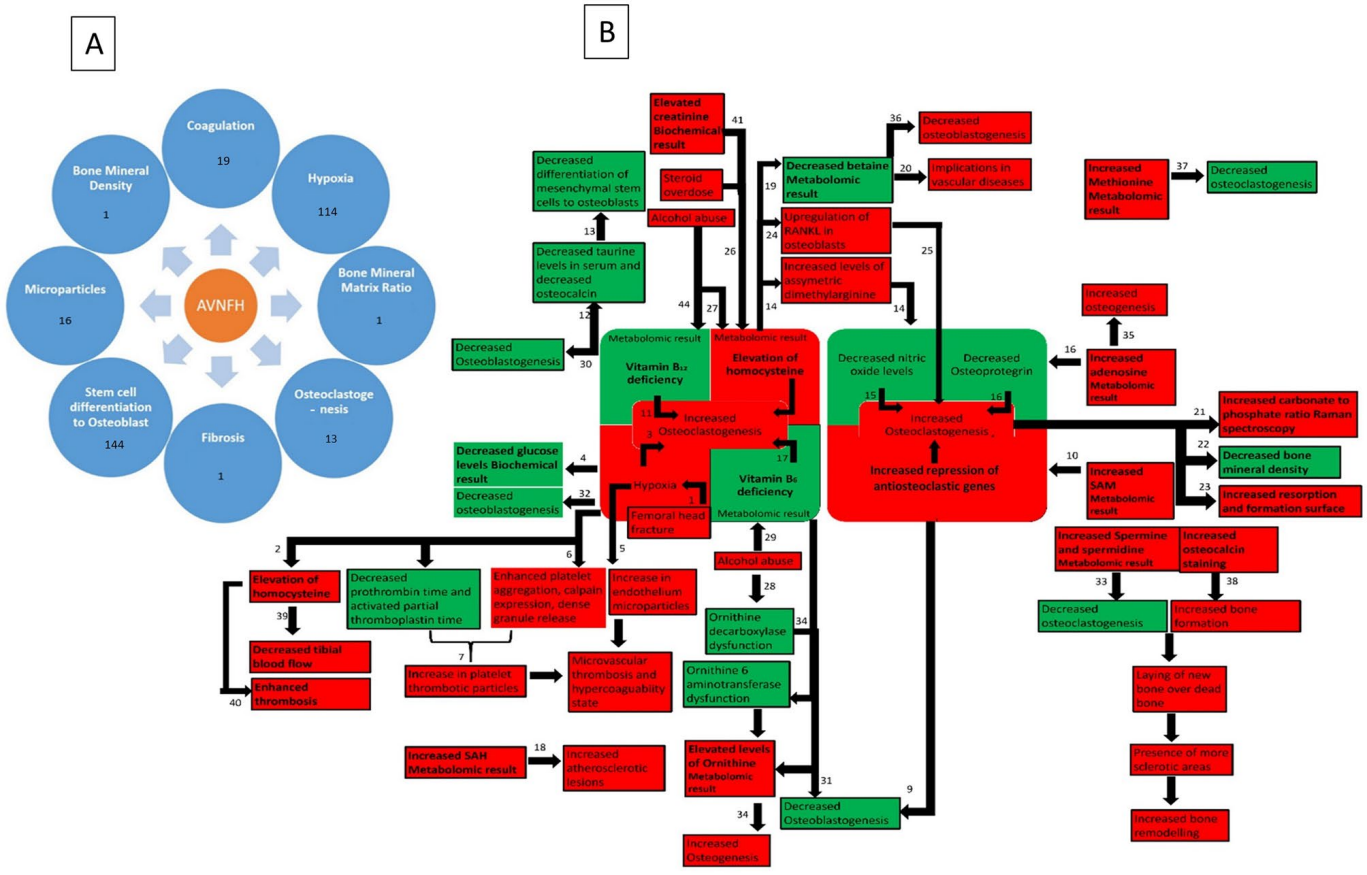
AVNFH as a multi systemic disease. (**A**) Different processes implicated in AVNFH from literature. (**B**) Possible pathogenesis model of AVNFH constructed on the basis of biochemical, omics, biophysical results and literature mining (references are provided in supplementary references S7).

Over all the integrative analysis not only agrees with the results of individual pathways but also provided important pathways that are common to AVNFH from different studies. Our results show the enrichment of genes in pathways that describe the symptoms and its associations with AVNFH such as elevation in homocysteine, coagulation, hypoxia, Vasoconstriction, endochondral ossification, osteoclastogenesis and their eventual effect (Fig. 6). Above all, the observations of Micro-Raman spectroscopy, Histopathology, CT, IHC and MRI studies correlate with the molecular signatures obtained from the Omic analysis. A summary of the role of different pathways and possible processes which they influence in AVNFH is provided (Fig. 7 and Supplementary Table S7.4).

### Analysis of 125 genes common to at least two data sets from Integrative analysis reveals their association with many diseases which are risk factors for AVNFH

**Figure 8:**
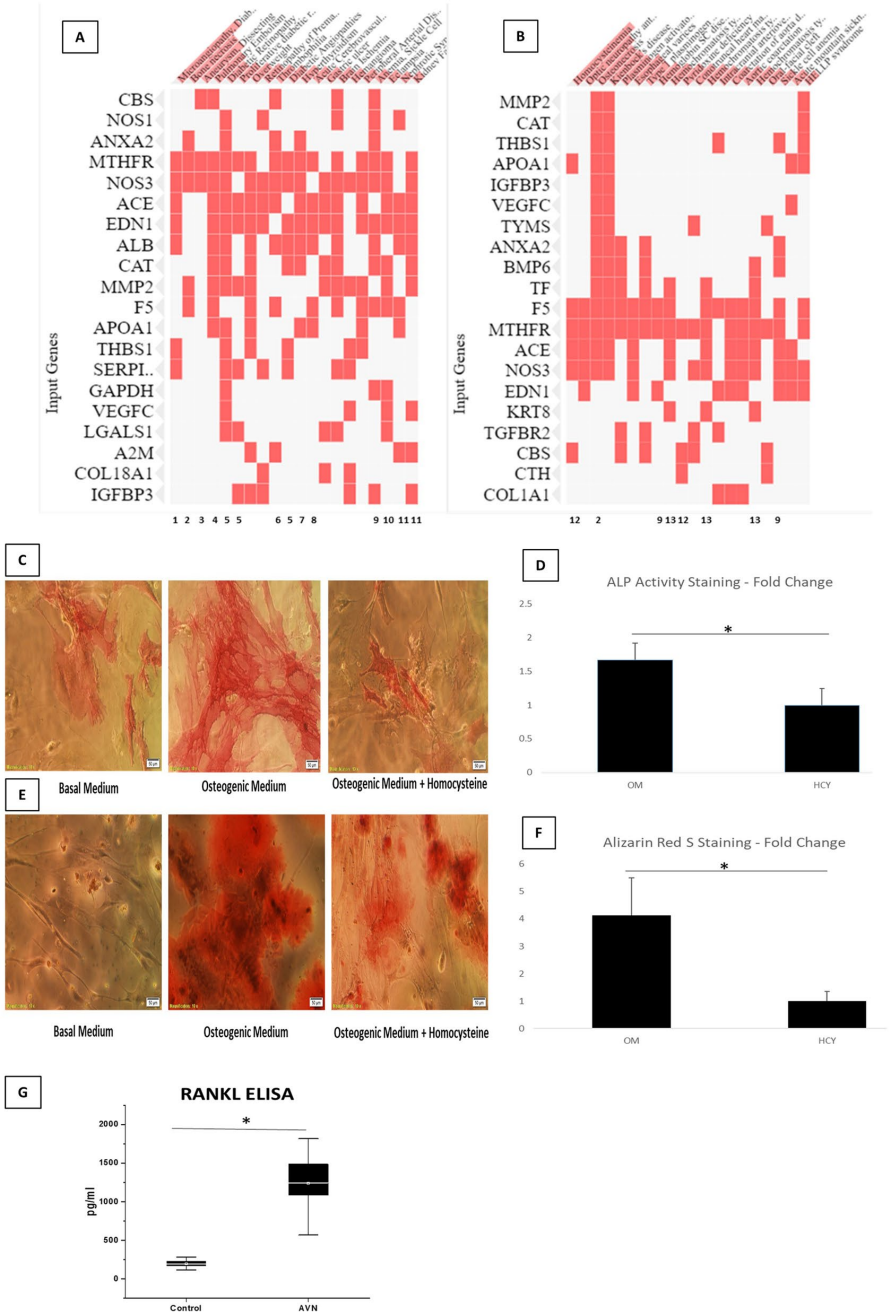
(**A**) DisGeNet clustergram showing enriched disease terms and associated genes. Numbers below show the reference which shows association of the disease with AVNFH. (**B**) Rare diseases GeneRIF genelists clustergram showing enriched disease terms and associated genes. Numbers below show the reference which shows association of the disease with AVNFH. (**C**) ALP activity staining showing reduced ALP activity (ALP staining) during osteogenic differentiation, in presence of Homocysteine. (**D**) ALP activity Staining quantification using ImageJ (Version: 1.46r URL: https://imagej.nih.gov/ij/) (P. value = 0.03) (**E**) Alizarin Red S staining showing reduced matrix mineralization during osteogenic differentiation in presence of Homocysteine. (**F**) Alizarin red staining quantification (P. value = 0.02). (**G**) Box plot showing elevated levels of RANKL in AVNFH patients (with high homocysteine, Narayanan et al. 2017) compared to healthy controls (P. value = 0.0074). P. value ≤ 0.05 was considered to be significant.

The integrative analysis using data sets from SNP, Transcriptomics, proteomics and enzyme-cofactor data showed 125 genes to be common to at least two data sets. These 125 genes were used for further analysis for diseases enrichment employing DisGeNET and Rare diseases gene set libraries from Enrichr. The top 40 genes were found to be associated with various diseases (Fig. 8A,B). These diseases include Microangiopathy, bone necrosis, aneurysm, pulmonary embolism, diabetic angiopathies, overweight, thrombophilia, hyperthyroidism, cerebrovascular events, anemia, sickle cell eclampsia, nephrotic syndrome, homocysteinemia, Kidney diseases, hemochromatosis and other diseases (Fig. 8A,B). Metabolomic analysis of perfused renal cortex samples from diabetic kidney disease rat models showed derangement of metabolites belonging to methionine pathway^36^. Further, by literature mining we could show that most of the diseases found using DisGeNET analysis were found to be risk factors for AVNFH (Fig. 8A,B). Taken together our analysis with molecular signatures could predict the diseases which are risk factors associated with AVNFH.

### Validation of homocysteine and osteoclastogenesis (RANKL) module by experiments and literature mining

The analysis carried out in this study shows SNP in MTHFR, low B_6_, B_12_ and Folate as well as factors like Alcoholism by literature mining to be associated with AVNFH. The data analysis using SNP, transcriptomic, proteomic and metabolomic and the enzyme-cofactor data also showed involvement of elevated homocysteine in AVNFH (Fig. 6). Since the etiological, genetic and metabolic factors lead to elevated levels of homocysteine, we treated Mesenchymal Stem Cells with basal medium as well as osteogenic medium with and without homocysteine. MSC treated with homocysteine led to a significant reduction in ALP staining indicative of reduced osteoblastogenesis (Fig. 8C,D). Further mineralization assay using alizarin red showed significantly reduced mineralization in homocysteine treated sets compared to controls (Fig. 8E,F).

Systems analysis using the data sets shows osteoclastogenesis as one of the modules modulating the disease process (Fig. 6). RANKL is involved in osteoclastogenesis. Since SNP in RANKL is associated with AVNFH and transcriptomic analysis of AVNFH showed RANK-RANKL signaling, we estimated the level of RANKL in AVNFH patients who had high homocysteine^1^ compared to controls. RANKL was found to be significantly elevated in AVNFH patients compared to controls (Fig. 8G). Our integrative analysis showed CBS and MUT to be common to three of the four data sets used. Hence, we carried out literature mining to see if knock down, knock out, cofactor depletion or supplementing downstream products of CBS could modulate RANKL induced osteoclastogenesis. The results shows that depletion of B_6_ and B_12_ significantly augmented RANKL induced osteoclastogenesis (Supplementary Table S7.7). RANKL treatment in presence of siRNA which knock down CBS also significantly increased osteoclastogenesis (Supplementary Table S7.7). Similarly RANKL in presence of homocysteine significantly elevated osteoclastogenesis (Supplementary Table S7.7). Further, RANKL induced osteoclastogenesis could be inhibited by treatment with glutathione (Supplementary Table S7.7). Mice model of CBS KO exhibited hyperhomocystenemia and osteoporosis. Supplementation with N-Acetylcysteine was shown to decrease osteoclastogenesis and increase bone mass (Supplementary Table S7.7). Mice model of MUT KO exhibited hematological abnormalities like mild macrocytic anemia and low bone mineral density (Supplementary Table S7.7). Depletion of Vitamin B_12_ was shown to stimulate osteoclastogenesis by way of increasing homocysteine and methyl malonic acid (Supplementary Table S7.7).

Taken together our –Omic analysis results as well as integrative analysis using different data sets shows CBS and MUT to be associated with the disease. CBS and MUT, for their activity require B_6_ and B_12_ as cofactors respectively, which is present in low levels in the patients. Knock down, knock out or cofactor depletion (B_6_, B_12_) lead to elevated levels of homocysteine and methyl malonic acid which modulate MSC differentiation to osteoblasts, macrophage differentiation to osteoclasts as well as their function with potential implications for the disease.

## Discussion

SNPs associated with AVNFH are collected from the literature. Network of SNPs associated with AVNFH was generated using Cytoscape and the SNPs in genes were binned into pathways. Furthermore, the functional implications of SNPs that were validated experimentally was collected from literature and its implication for AVNFH was discerned. The SNPs were associated with elevated homocysteine, hypoxia, osteoclastogenesis or coagulation and vasoconstriction. Transcriptomic analysis binned the genes into signaling pathways, metabolic pathways and other pathways that has implication for bone biology and disease manifestation. Proteomic data collected from the literature shows changes in pathways like B_12_ and coagulation which has implications for disease process. The cofactor-enzyme network conjured pathways that are deregulated in the disease, which has major implications for disease progression. The integrated analysis of SNP, Transcriptomic, proteomic and cofactor network shows common pathways that are deregulated in the disease that are very critical to the disease process. Analysis of the genes that are common to at least two data sets in the integrative analysis using DisGeNET showed association with multiple diseases. Interestingly cross validation using literature mining showed that these diseases are risk factors associated with AVNFH. In order to validate the prediction of a role for elevated homocysteine in vitro studies were carried out using MSC. Experimental validation of the role of homocysteine shows impaired MSC differentiation to osteoblasts and their function. In addition to this we also probed into the role of RANKL in AVNFH by measuring the levels of RANKL in our patient cohort. Subsequently the factors that modulate RANKL induced osteoclastogenesis was validated by literature mining. Our analysis showed elevated levels of RANKL in AVNFH patients. Using literature mining we show that RANKL induced osteoclastogenesis is augmented by homocysteine, depletion of B_6_ and B_12_, knock down/knock out of CBS or MUT. RANKL induced osteoclastogenesis could be attenuated by supplementation of glutathione or N-Acetylcysteine in CBS KO mice. Taken together the analysis recapitulates the observed processes associated with the disease which could be validated by experiments and literature mining.

Previous studies have shown significant association of elevated levels of homocysteine with AVNFH^1^. The analyzed data at the level of SNPs, Transcriptomic, proteomic, metabolomics or their integrative analysis shows involvement of cysteine and methionine pathway, one carbon metabolism, B_12_ metabolism, B_6_ and folate metabolism, megaloblastic anemia etc. In addition, alcoholism which is a risk factor for AVNFH also leads to reduced levels of B_12_ and increased levels of homocysteine^37^. Our previous studies have shown lower levels of hemoglobin in AVNFH patients compared to age and gender matched controls^1^. However, whether the reduced hemoglobin correlated with megaloblastic anemia was not confirmed. Homocysteine has implications for bone biology as it remodels osteoblastogenesis, osteoclastogenesis and their function^38,39^. Homocysteine is also an inflammatory molecule and induces inflammatory response in the macrophages. Homocysteine is known to inhibit the activity of NOS. NOS is an important enzyme which is essential for vascular function^40^. Inhibition of NOS also leads to increased osteoclastogenesis^41^. Homocysteine also modulates blood coagulation^42^. In addition, homocysteine is known to induce ROS^43^. B_6_ is an important vitamin which is a cofactor for cystathionine beta synthase involved in the synthesis of cysteine and thus glutathione^44^. Reduced levels of B_6_ not only contribute to increased homocysteine but also to decreased cysteine, which is essential for the synthesis of glutathione. Our integrative analysis shows CBS as a key enzyme in the transsulfuration pathway which might be involved in AVNFH. Thus SNPs or vitamins like B_6_, B_12_ and folate or alcoholism might modulate the levels of homocysteine in different populations which in turn influences multiple pathways in the progression of AVNFH.

AVNFH has also been associated with coagulation disorders. Consistent with this SNP analysis Using the Plugin ClueGO binned genes into blood clotting cascade, complement and coagulation cascade. ClueGO analysis of network generated with the SNP containing genes along with first neighbors show that the genes are binned into Platelet degranulation pathway. ClueGO analysis of Network generated from Proteomic data sets shows genes binned into platelet degranulation, complement and coagulation cascade. Platelet degranulation is an important process in coagulation pathway^45^. Previous studies have shown an association of homocysteine with AVNFH^1^. Homocysteine potentiate ADP induced platelet degranulation and coagulation^46^. Increased Von Willebrand factor which is involved in coagulation process is also associated with AVNFH^47^. SNP in Collagen associated with AVNFH is shown to induce platelet activation^48,49^. In addition, microparticles in blood are associated with the disease^16^. The association of coagulation and vasoconstriction is also supported by histopathology and MRI-BOLD as well as blood velocity studies^50^. Hence multiple factors at the genetic, transcriptomic, metabolomic or at the level of cofactors might contribute to coagulation cascade which is associated with AVNFH.

Previous studies using Micro-Raman Spectroscopy shows increased carbonate to phosphate and decreased mineral matric ratio in AVNFH bone from both patients and piglet model^1,11^. CT scan shows reduced Houns field unit an indication of reduced bone density^1^. The data is suggestive of increased osteoclast and decreased osteoblast function. The increased resorption of bone is ascribed to increased osteoclastogenesis and its function^51^. In addition, AVNFH bone shows increased bone turnover as observed in the Immunohistochemistry (IHC) for osteocalcein^1^. Homocysteine is shown to modulate osteoclastogenesis. Studies have reiterated a role for homocysteine in osteoblast induced osteoclastogenesis by way of RANKL expression and secretion^32^. B_12_ and B_6_ depleted media are shown to augment the differentiation of macrophages to osteoclasts^52^. Our AVNFH patient cohort show elevated level of RANKL in plasma. IHC of patient bone also shows increased RANK, RANKL staining^13^. More so, Iron sequestration is an important event in osteoclastogenesis^53^. Consistent with these facts, RANKL induced osteoclastogenesis shows upregulation of genes involved in iron metabolism^54^. Knocking down genes involved in endosome acidification was shown to inhibit osteoclastogenesis^55^. SNP analysis shows HIF-1 signaling pathway and VEGF signaling pathway are associated with hypoxia. SNP and Transcriptomic analysis shows regulation of gene expression by HIF-1α. Traumatic AVNFH is associated with hypoxia as seen in animal model of AVNFH^56^. In addition, the microparticles in blood associated with ANVFH is proposed to clog capillaries inducing hypoxic conditions^16,17^. Consistent with this immuno-histochemistry shows increased HIF-1 staining in AVNFH bone^15^. MRI-BOLD technique also shows changes in the oxygenation of arterial and venous blood in AVNFH patients compared to controls (Supplementary Table S7.5 and references there in). Hypoxia is involved in osteoclastogenesis and lead to bone resorption^57^. Transcriptomic analysis shows TGFβ signaling pathway is important for osteoclastogenesis and TGFβ Receptor knock out inhibits osteoclastogenesis^58^. Thus multiple signaling pathways together might provide conditions favorable to osteoclastogenesis and bone degradation.

Homocysteine is shown to inhibit the expression of LOX1^59^ which is involved in collagen cross linking. Previous studies have shown Homocysteine inhibit the differentiation of mesenchymal stem cells (MSC) into osteoblasts^60^. However, contradictory results are obtained in case of homocysteine induced mineralization^61^. Increased iron also inhibit differentiation of MSC into osteoblasts^62^. Consistent with this a role for iron is conceived in osteoclastogenesis, osteoblastogenesis and their function. Iron overload diseases like sickle cell anemia, beta-thalassemia and hemochromatosis are risk factors for AVNFH. The histopathological changes also report empty lacunae in bone, indicative of absence of osteoblasts, reverse cement lines, marrow calcification etc., which shows deregulation and imbalance in osteoblast and osteoclast function^1^. The different pathways might act in concert to impede MSC differentiation into osteoblasts and promote differentiation of macrophages into osteoclast as well as modulate their function.

Endochondral ossification is a process where cartilage is replaced by bone^63^. Network analysis of SNPs associated with AVNFH and transcriptomic analysis of AVNFH cartilage shows binning of genes involved in endochondral ossification pathway. Cartilage in AVNFH shows hypertrophy and death in AVNFH^64^.

Treatment with Bisphosphonate is the standard care treatment for AVNFH^65^. Bisphosphonate acts at multiple levels by inhibiting osteoclastogenesis and preventing coagulation by inhibiting platelet degranulation^66^. Bisphosphonate also helps in increasing mineralization^65^. However, in the absence of osteoclastogenesis, this might lead to woven bone formation which might hamper bone strength^67^.

In the present study, analysis of genes common to at least two data sets using DisGeNET showed association with various diseases. Validation using literature showed these diseases are risk factors associated with AVNFH (Supplementary Table S7.6 and the references there in). However, in the diseases which are risk factors for AVNFH, if the associated genes of our DisGeNET analysis are involved in disease process leading to AVNFH remain to be experimentally validated. Analysis of SNPs, the expression levels of these genes or levels of cofactors required for their activity might shed light on their role in progression to AVNFH.

Further, our work shows that elevated levels of homocysteine ensue, as a consequence of multiple factors like SNP on MTHFR, reduced B_6_, B_12_ or folate. We show that elevated Homocysteine leads to impairment of MSC differentiation into osteoblasts and inhibit their mineralization function. Previous studies have also shown that homocysteine causes apoptosis in mesenchymal stem cells^60^. Transcriptomic analysis shows RANK-RANKL signaling in AVNFH. We show that RANKL is elevated in the plasma of AVNFH patients. Literature mining shows that RANKL induced osteoclastogenesis is augmented by depletion of B_6_ or B_12_ in media. Knock down of CBS which came up as a critical gene in three of the four data set in our integrated analysis increased osteoclastogenesis, while supplementation of glutathione was shown to inhibit osteoclastogenesis^68^. CBS KO mice showed increased osteoclastogenesis and osteoporosis which could be mitigated by supplementation of N-acetyl cysteine. Similarly, MUT KO mice exhibited anemia and reduced bone density^69^.

Overall the results of our analysis show that the molecular signatures of SNPs, transcriptomics, proteomic, metabolomic and cofactor-enzyme data captures the tenets of pathophysiology and symptom associated changes in bone biology, biophysical, immunohistochemical and histopathological changes characteristic of the disease. However, due caution should be exercised as the work with human and animal model systems only can be used to reiterate the conserved pathways as the animal models may not be truly representative of AVNFH in humans. This work also paves way for a deeper insight into the role of homocysteine and iron metabolism in the pathophysiology of AVNFH. The work also shows various diseases which are risk factors associated with AVNFH, and raises additional questions on the potential role or association of different genes in AVNFH. The work also helps to delineate the potential benefits of treatment with bisphosphonate as it interferes with ostoclastogenesis and coagulation process associated with AVNFH.

## Conclusions

Our integrative analysis points to the contribution of multiple factors like hypoxia, coagulopathy, deficiency of vitamins like B_6_, B_12_, folate, high homocysteine, osteoblastogenesis, osteoclastogenesis, endochondral ossification etc., in remodeling of AVNFH bone and progression of the disease. The molecular signatures correlate with observations like coagulation, hypoxia, decreased osteoblastogenesis, increased osteoclastogenesis and their deregulated function in the disease. This in turn complies with previous MRI, biophysical, immunohistochemical and histopathological studies of the AVNFH bone. The role of coagulation pathway in the diseases and factors that affect it also are evident from the systems analysis of data sets which might contribute indirectly to disease process. The work also shows multiple factors like SNPs, Alcoholism, cofactor deficiency or iron overload diseases as well as other diseases that could lead to similar environment which might favor disease progression. A role for specific genes involved in these diseases which are risk factors for AVNFH remain to be ascertained experimentally. The present work also provides direction where supplementation of vitamins like B_6_, B_12_ and folate might help in management of the disease. The result of our analysis shows AVNFH as a multi-systemic disease.

## Material and methods

### SNPs associated with AVNFH and functional implication

The SNPs associated with AVNFH were obtained from literature. The inclusion criteria were all SNPs that have association with AVNFH/osteonecrosis, while all those that did not show any association in a particular study with AVNFH/osteonecrosis were excluded. GWAS data for AVNFH/osteonecrosis was also searched and SNPs were included. SNPs which were associated only with osteonecrosis other than that of femoral head were excluded. The SNPs were categorised into Exonic, which contained the synonymous and non-synonymous SNPs, Intronic in introns, promoters, 3ÚTR and intergenic regions. The list of SNPs was binned into pathways using ClueGO 70 plugin of cytoscape for pathway annotation 71 and the pathway networks were visualised in Cytoscape. ClueGO uses KEGG, Reactome and WikiPathways 72–74 to bin genes into pathways. Further the functional implication of SNPs was discerned by literature search for experimentally validated data. For finding experimental validation of SNPs associated with AVNFH from literature OMIM data base was searched and search was also carried out with rs ID or name/notation of genes followed by functional implications or experimental validation. All SNPs with impaired function or implications for a function were included while those without any consequence were excluded.

### AVNFH gene expression microarray, proteomic and metabolomics data

The gene expression microarray study involved a genome wide Gene expression profiling of hip articular cartilage with AVNFH. Gene expression microarray data of GEO accession GSE74089 was obtained from Gene Expression Omnibus^75^ (GEO; https://www.ncbi.nlm.nih.gov/geo/) based on the platform GPL13497 (Agilent-026652 Whole Human Genome Microarray 4 × 44 K v2), deposited by Ruiyu L^24^. Gene expression profiling of AVNFH articular cartilage was carried out by collecting hip articular cartilage specimens from 12 AVNFH patients and 12 healthy controls. Microarray data of GEO accession GSE74847 was used which studied RANKL induced osteoclastogenesis using RAW 264.7 cells^76^. The Proteomic data from AVNFH bone tissue compared to healthy control bone tissue was used from a published dataset^26^. Data from three published metabolomic analyses were used for our study. Our previous study involving targeted metabolomics of plasma samples from 14 patients compared to 14 healthy control samples were used^1^, a global non-targeted metabolomics of plasma samples from 30 AVNFH patients compared to 30 healthy controls^23^, and a bone trabecular metabolomics study of 28 patients compared to 20 controls^25^.

### Differential gene expression analysis and data pre-processing

The GEO2R web tool was used to define two groups of samples namely (necrotic femoral head) ‘NFH’ and ‘Control’, and to perform Differential Gene Expression analysis^77^. The GEO2R tool uses the GEO-query and limma R packages from the Bioconductor project to compare the original processed data tables supplied by the submitter. The GEOquery R package parses GEO data into R data structures that can be used by other R packages^77^. The limma (Linear Models for Microarray Analysis) R package has statistical tests for identifying differentially expressed genes^78^. The GEO2R tool was used to define groups such that the logFC gave fold change of NFH group with respect to Control group. i.e., a positive logFC implied upregulation of gene in NFH group and negative logFC implied downregulation in NFH group.

The adjusted p. Value from the Limma package was used to identify genes that were significantly differentially expressed between the Control and NFH patient sample. The Value adjustments, also called multiple-testing corrections, attempt to correct for the occurrence of false positive results. The Benjamini & Hochberg false discovery rate method^79^ was used for adjustment of the microarray data and provide a good balance between discovery of statistically significant genes and limitation of false positives. Significant differentially expressed genes of Adjusted P. Value ≤ 0.05 were used for this study. Significant changes in the proteomic and metabolomics analyses were obtained from the respective published datasets.

### ClueGO and metaboanalyst pathway annotation analyses

The ClueGO plugin of Cytoscape was used for pathway annotation analysis^70,71^. ClueGo can perform Enrichment (right-sided hypergeometric test), Depletion (left-sided hypergeometric test) or Enrichment/Depletion (two-sided hypergeometric test). We have used Enrichment/Depletion (two-sided hypergeometric test) for our analyses which are recommended^80^. The significant differentially expressed genes were used for querying Reactome, KEGG and WikiPathways^72–74^ databases for pathway functional analyses. The pathway terms showing term P. Value ≤ 0.05 were considered for further analysis. Significant pathways of interest were used for creating subnetworks containing specific daughter pathways with genes involved to understand differential gene expression and predict mechanism of derangement of metabolic pathways. Cytoscape tools were used to generate specific pathway networks from global pathways.

Cluepedia plugin of Cytoscape was used to add all genes present in all the pathway terms of the network^81^. Subnetworks of pathways showing pathway terms and their associated genes were created. logFC data of genes was then imported into cytoscape and was overlaid with the subnetwork. MetaboAnalyst tool was used to bin significant metabolites into KEGG Pathways^30^.

### Vitamin B_6_, vitamin B_12_ and folate cofactor: protein interaction network

Since Vitamin B_6_, B_12_ and Folate cofactors are implicated in AVNFH^1,82^, an interaction network involving these cofactors and respective proteins was created and visualised in Cytoscape^71^. We have created this network based on the published dataset involving cofactors–protein interaction network used for understanding relation between human nutrition and diseases^31^. The created Vitamin B_6_, B_12_ and Folate cofactors – protein network was used for ClueGo analyses.

### Integratedomic network construction and network centrality analyses

Networks for individual analyses involving AVNFH SNPs, Proteomic, Transcriptomics and Cofactor-protein networks were created using Cytoscape. Next these individual networks were merged in Cytoscape to obtain an integrated network. To understand common key nodes in AVNFH, network centrality analysis was carried out using CytoNCA plugin of Cytoscape 83. The hub nodes obtained from network centrality analysis was used for further functional analyses using ClueGO.

### Drug/disease enrichment analyses using Enrichr

The 125 hub nodes from integrated omics network with degree 2 and 3 were used to carry out Drug/Disease enrichment analyses. Enrichr web—based tool was used to carry out Drug / Disease enrichment analyses 84. We used gene—set libraries from DisGeNet and Rare Diseases GeneRIF Gene Lists. DisGeNet is a comprehensive repository of human gene—disease associations. Rare Diseases GeneRIF Gene Lists has gene—disease associations based on GeneRIF (Gene Reference into Function) to enrich the functional annotation of genes. The enriched terms were sorted using combined score ranking after carrying out the enrichment analyses using Enrichr tool.

### RANKL ELISA

All the blood samples of AVNFH (n = 5) and controls (n = 7) were procured from patients visiting Sri Sathya Sai Institute of Higher Medical Sciences in a de-identified manner by a honest broker as per approval of the SSSIHMS institutional bioethics commission (Approval number: SSSIHL/IEC/PSN/BS/2012/05) Informed consent was obtained from all subjects and the methods were carried out in “accordance” with the approved guidelines and regulations. Plasma samples from healthy control, and AVNFH were frozen at − 80 °C until assay was done. Commercial ELISA kits (Peprotech) of human sRANK ligand (Cat. No. 900-M142), were used in the study as per manufacturer’s instructions. Dilutions of antibodies were carried out as per manufacturers instruction unless otherwise specified. Briefly, 96 well micro-titer plates (Corning Product #3590) were coated with capture antibody and incubated overnight at room temperature. Following this, the plate was first washed with wash buffer and blocked with block buffer for 1 h at room temperature. Standards and sample dilutions were prepared using the diluent and pipetted into designated wells. Detection antibody was added immediately into the standards and sample wells and incubated for 2 h at room temperature. After thorough washing, Avidin- HRP conjugate was added and incubated for 30 min at room temperature. Finally, ABTS Liquid substrate provided by the manufacturer in the respective kit was added into each well and incubated for 30 min at room temperature. Absorbance at 405 nm (reference absorbance 650 nm) was obtained within 30 min of adding the stop solution and the results were calculated using a log–log or 4- parameter curve fit. P. value ≤ 0.05 was considered significant.

### MSC isolation from mice

Animal experiments were approved by the Institutional Animal Ethics Committee, Indian Institute of Science, Bangalore, India. All animal protocols were performed in accordance with the guidelines for care and use of laboratory animals set by Indian National Science Academy. Six to eight -week-old CD-1 female mice, weighing 25–28 g, were used for the isolation of BM-MSCs BM-MSCs were isolated as previously described (PMID: 29760732). Briefly, the mice were sacrificed by cervical dislocation and the femurs and tibias were dissected out. The bone marrow was flushed with Dulbecco's phosphate-buffered solution (DPBS; Thermofisher scientific, Waltham, USA). The cell suspension was filtered through a 70 μm cell strainer (BD Falcon, USA), and centrifuged at 300×*g* for 10 min. The cell pellet was suspended in 1 ml MSC culture medium composed of Dulbecco's modified Eagle's medium (DMEM) supplemented with 10% fetal bovine serum (FBS), 1% penicillin/streptomycin and 2 mM glutamine (all purchased from Thermo Scientific, Waltham, USA). Cells were seeded in 35 mm cell culture dishes at a density of 1 × 106 cells/cm2 and incubated in a humidified incubator at 37 °C, 5% CO_2_. After 1 day, nonadherent cells were removed by washing twice with DPBS and fresh MSC culture medium was added. The medium was replaced with fresh MSC culture medium every 2 days.

### MSC cell culture and osteogenic differentiation

Mouse bone marrow derived Mesenchymal Stem Cells (MSCs) were cultured in basal medium (BM) consisting of DMEM (Gibco) supplemented with 10% FBS (Gibco), 1% Penicillin –Streptomycin solution (Gibco) and 1% Glutamax (Gibco). Cells were cultured in 4 well plates in basal medium till cells reached 70% confluence following which the basal medium was replaced with Osteogenic medium (OM) which consisted of basal medium supplemented with Ascorbate—100 µM (Sigma-Aldrich) and β-glycerophosphate—5 mM (Sigma-Aldrich). During osteogenic differentiation, effect of appropriate concentration of Homocysteine—30 µM^60^ was studied by using DL Homocysteine (Sigma-Aldrich). Medium was changed every 3 days.

### ALP activity staining and Alizarin Red S staining

Effect of Homocysteine on Alkaline Phosphatase activity during osteogenic differentiation was carried out after 7 days of treatment. ALP activity staining was carried out by fixing cells with 4% para formaldehyde. After fixation the cells were stained with staining solution containing Napthol–AS–MS Phosphate—0.4 mg/ml (Sigma-Aldrich), Fast Red TR salt—1 mg/ml (Sigma-Aldrich), dissolved in Tris-Maleate buffer—30 mM at pH 9, in presence of 0.08% MgCl2 solution. At pH 9, in presence of Mg2 + and Alkaline Phosphatase, Napthol–AS–MS Phosphate and Fast Red TR combine to form an insoluble Azo red end product.

Effect of Homocysteine on matrix mineralization, was carried out using Alizarin Red S staining to stain calcium deposits. After 14 days of treatment, the cells were fixed with 4% para formaldehyde. Then the cells were stained with 2% Alizarin Red S solution (Sigma-Aldrich) whose pH was adjusted to 4.2 using ammonium hydroxide solution. Alizarin Red S stains calcium deposits to give red colouration.

ALP activity staining and Alizarin staining was quantified using ImageJ software (Version: 1.46r URL: https://imagej.nih.gov/ij/), by calculating the intensity of the stained area. Data is presented as mean fold change of at least 5 sample images from each group. P. Value ≤ 0.05 was considered significant.

### Ethics approval

Experiments on humans and the use of human blood samples: We confirm that all experiments were performed in accordance with relevant guidelines and regulations.

## Supplementary information

Supplementary Information 1.

Supplementary Information 2.

Supplementary Information 3.

Supplementary Information 4.

Supplementary Information 5.

Supplementary Information 6.

Supplementary Information 7.
