# Supplementary material for: Systems analysis of avascular necrosis of femoral head using integrative data analysis and literature mining delineates pathways associated with disease
Source: Sci Rep. 2020 Oct 22;10:18099. doi: 10.1038/s41598-020-75197-0 (PMC7581770; doi:10.1038/s41598-020-75197-0)
Supplement: Supplementary file 7 — Supplementary Information 7. [file 41598_2020_75197_MOESM7_ESM.docx]

**Systems analysis of Avascular Necrosis of Femoral Head using integrative data analysis and literature mining delineates pathways associated with Disease**

Ashwin Ashok Naik^1 ,^**^#^**, Aswath Narayanan^1,^**^#^**, Prakash Khanchandani^2^*, Divya Sridharan^3^, Piruthivi Sukumar^4^, Sai Krishna Srimadh Bhagavatam^1^, Seshagiri Polani^3^, Venketesh Sivaramakrishnan^1,^*

1. Disease Biology Lab, Dept. of Biosciences, Sri Sathya Sai Institute of Higher Learning, Prasanthinilayam, Andhra Pradesh-515 134.
2. Dept. of Orthopedics, Sri Sathya Sai Institute of Higher Medical Sciences, Prasanthigram, Andhra Pradesh-515 134.
3. Molecular Reproduction and Developmental Genetics, Indian Institute of Science, Bangalore
4. Leeds Institute of Cardiovascular and Metabolic Medicine, School of Medicine, University of Leeds, Leeds, LS2 9JT, United Kingdom.

**^#^**Both the authors contributed equally

*****To whom correspondence has to be send: Dr. Venketesh Sivaramakrishnan [svenketesh@sssihl.edu.in](mailto:svenketesh@sssihl.edu.in), [s.venketessh@gmail.com](mailto:s.venketessh@gmail.com), and Dr. Prakash Khanchandani [drprakashk@hotmail.com](mailto:drprakashk@hotmail.com), [prakash.k@sssihms.org.in](mailto:prakash.k@sssihms.org.in)


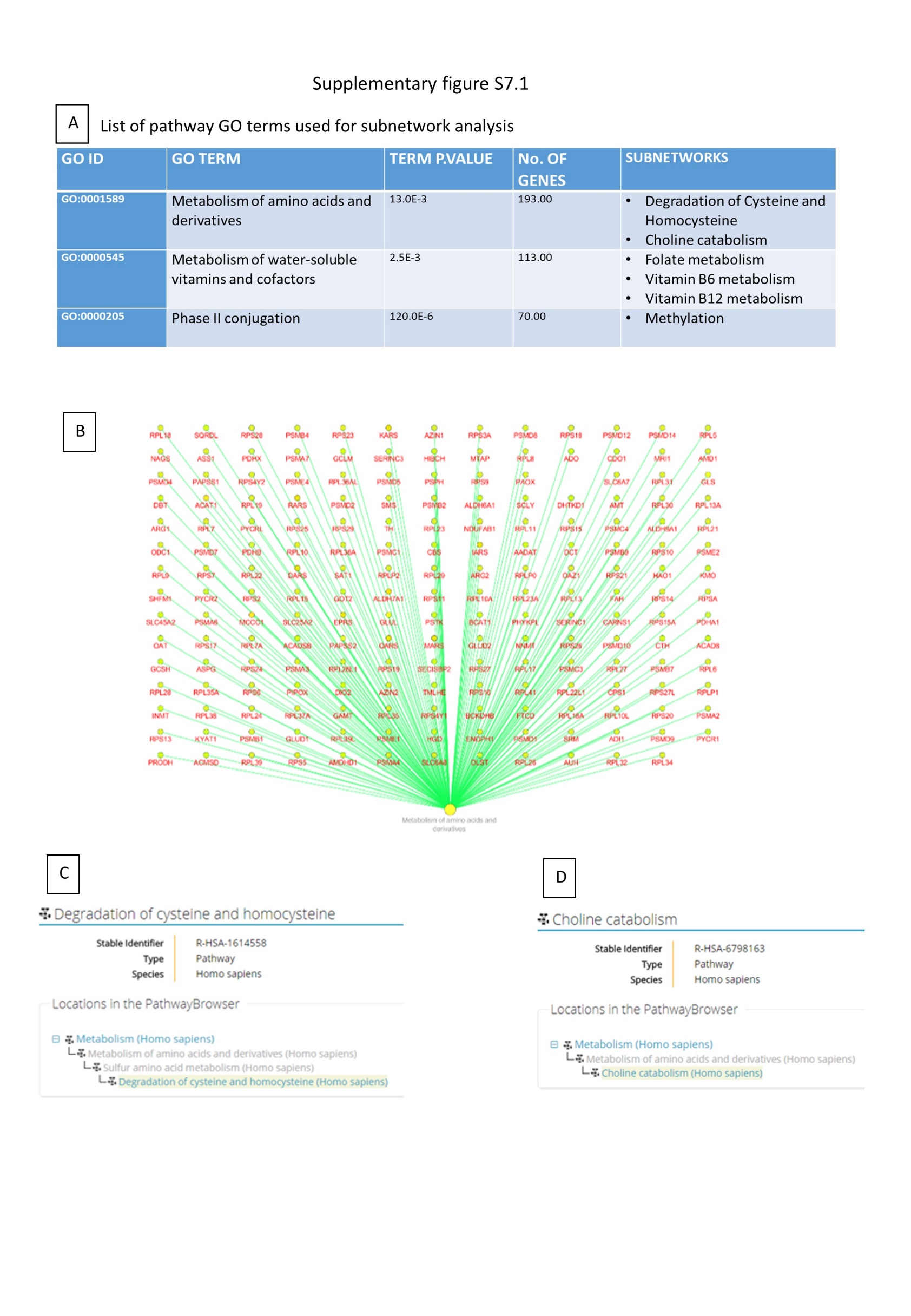


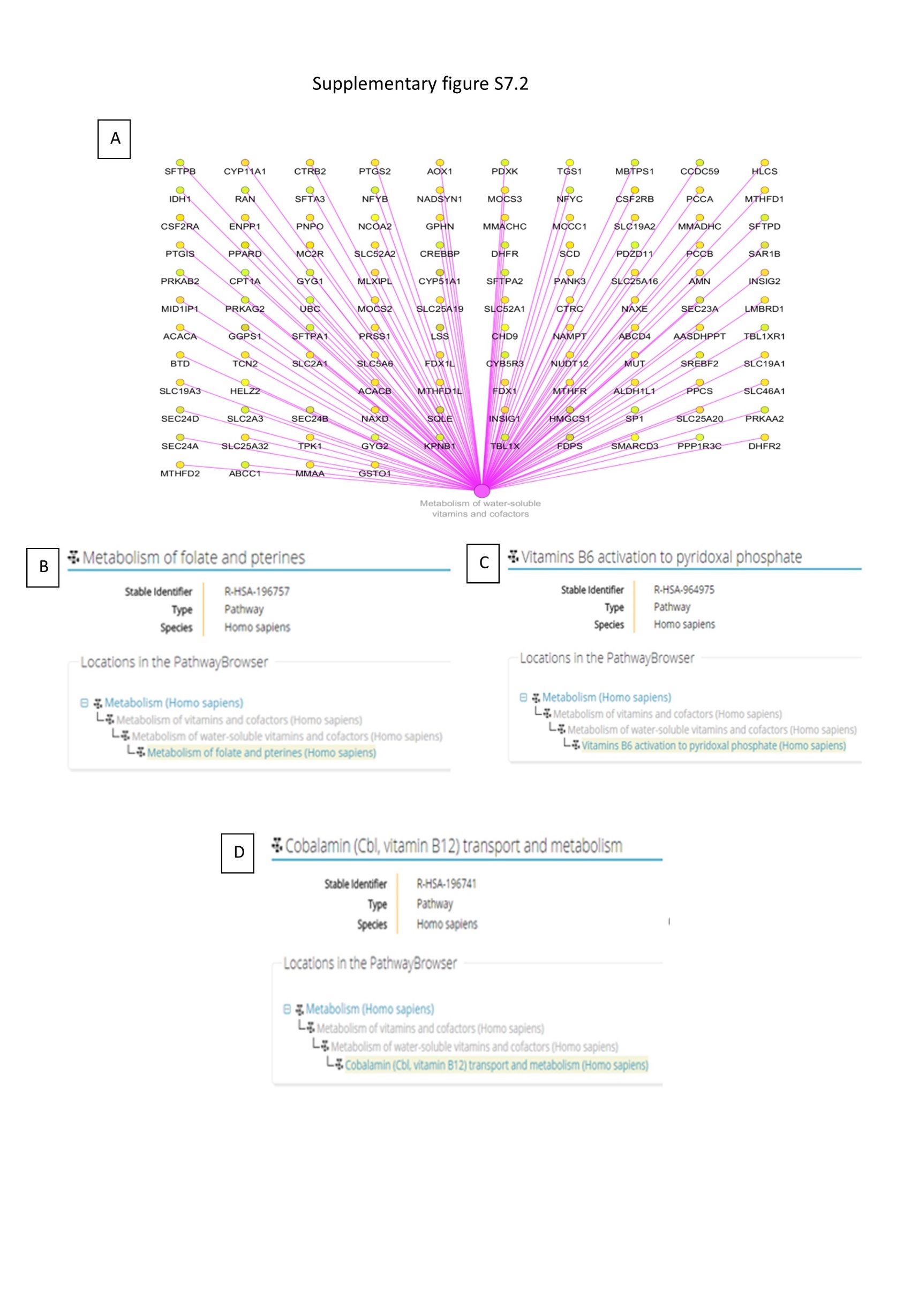


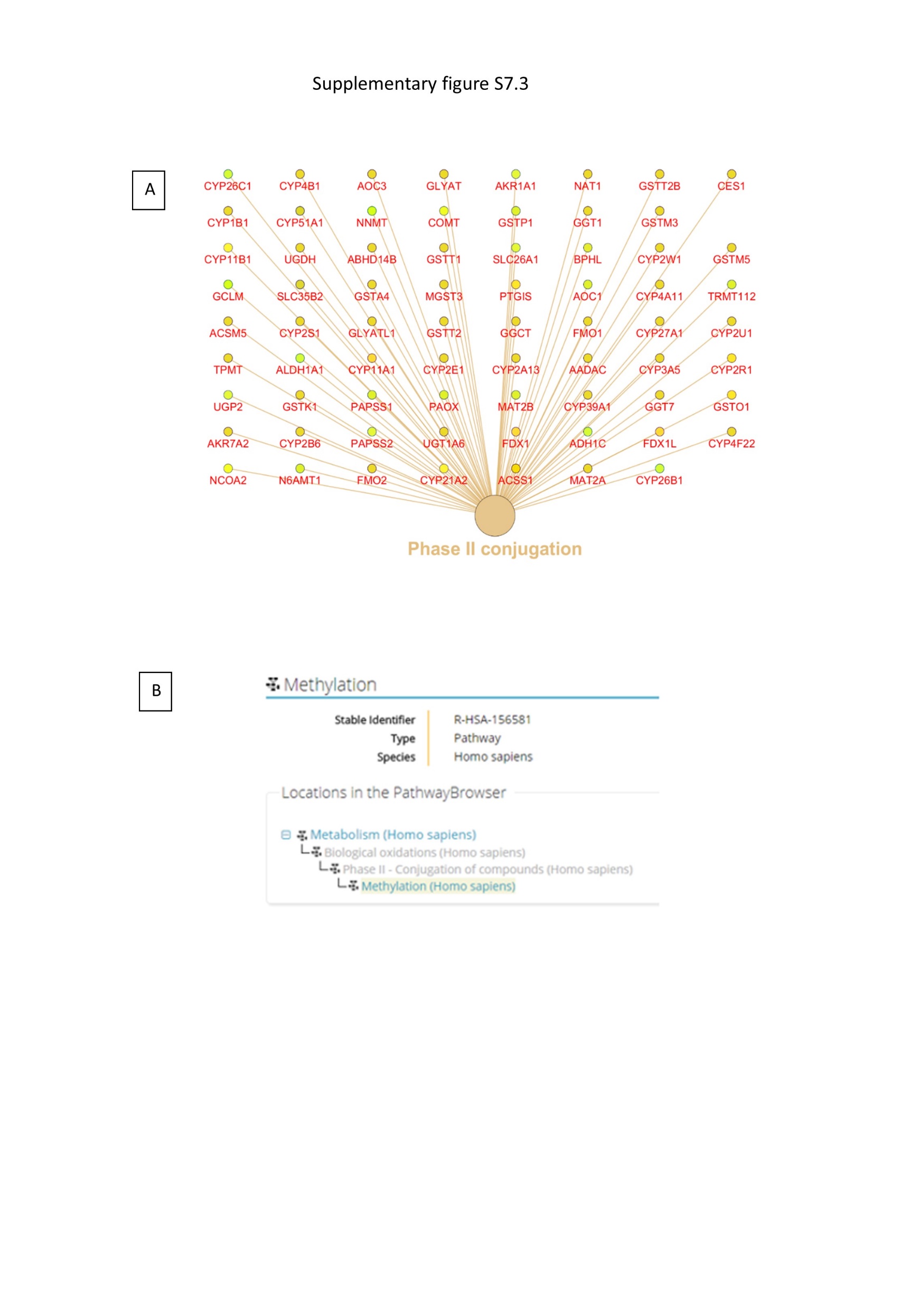


**Supplementary Table S7.4**: References legend for figure 7

| **Reference Number** | **Reference Details** | **Reference Number** | **Reference Details** |
| --- | --- | --- | --- |
| 1 | (Clayer and Bruckner, 2000) ^145^ | 23 | (Boyde and Hobdell, 1968) ^146^ |
| 2 | (Blaise et al., 2005) ^147^ | 24 | ^32^ (Reference from Manuscript) |
| 3 | (Arnett et al., 2003) ^148^ | 25 | ^32^ (Reference from Manuscript) |
| 4 | (Chen et al., 2007) ^149^ | 26 | (Nussinovitch, 2017) ^150^ |
| 5 | (Vince et al., 2009) ^151^ | 27 | (Medici et al., 2010) ^152^ |
| 6 | (Tyagi et al., 2014) ^153^ | 28 | (Lu et al., 2000) ^154^ |
| 7 | (Italiano Jr et al., 2010) ^155^ | 29 | (Lumeng and Li, 1974) ^141^ |
| 8 | (Dmitrieva and Burg, 2014) ^156^ | 30 | (Carmel et al., 1988) ^157^ |
| 9 | (De Vernejoul et al., 1984) ^158^ | 31 | (Holstein et al., 2009) ^159^ |
| 10 | (Nishikawa et al., 2015) ^160^ | 32 | (Utting et al., 2006) ^161^ |
| 11 | (Vaes et al., 2009)^162^ | 33 | (Yamamoto et al., 1988) ^163^ |
| 12 | (Roman-Garcia et al., 2014) ^164^ | 34 | (Tsai et al., 2015) ^165^ |
| 13 | (Zhou et al., 2014) ^166^ | 35 | (Takedachi et al., 2012) ^167^ |
| 14 | (Stühlinger et al., 2001) ^40^ | 36 | (Villa et al., 2017) ^168^ |
| 15 | (Kasten et al., 1994) ^41^ | 37 | (Vijayan et al., 2014) ^169^ |
| 16 | (Teramachi et al., 2011) ^170^ | 38 | (Tera et al., 2014) ^171^ |
| 17 | (Herrmann et al., 2009) ^172^ | 39 | (Tyagi et al., 2011)^38^ |
| 18 | (Kerins et al., 2001) ^173^ | 40 | (Lentz, 2005) ^42^ |
| 19 | (Imbard et al., 2015) ^174^ | 41 | (Piek et al., 1996) ^175^ |
| 20 | (Lever et al., 2005) ^176^ | 42 | (Xiao et al., 2015) ^177^ |
| 21 | (Aruwajoye et al., 2015) ^178^ | 43 | (Baggott and Tamura, 2015) ^179^ |
| 22 | (Conn, 2017) ^180^ | 44 | (Cravo et al., 1996) ^37^ |

**Supplementary Table S7.5**

| **Micro-Raman Spectroscopy** | |
| --- | --- |
| **Parameter and its inference** | **Description of Molecular signatures correlating with the inference of the parameters** |
| Mineral-Matrix ratio:  decreased (Human) ^1^  Reduced ostoblastogenesis and function | Functional implication of SNPs from literature resulting in elevated homocysteine and enhanced osteoclastogenesis (Figure 1D), Pathway annotation analysis of AVNFH patient cartilage transcriptomics showing the involvement of cysteine and homocysteine pathways (Figure 2E) and finally resulting in homocysteine accumulation (Figure 2G), Pathway annotation analysis of Vitamin B_6_, B_12_ and folate co-factor dependent protein network showing the involvement of cysteine, homocysteine and one carbon metabolism (Figure 4B and C), Analysis of integration of various -Omic analyses reveals involvement of increased Osteoclastogenesis (Figure 6B)  Increased homocysteine in AVNFH patients blood plasma (Metabolomics) ^1^, Homocysteine inhibits differentiation of mesenchymal stem cells (ALP staining, Figure 8C) and mineralization by differentiated osteoblast (Alizarin red staining, Figure 8E), Homocysteine inhibits the process of collagen cross linking by inhibiting the enzyme lysyl oxidase (LOX1) ^59^ |
| Carbonate to Phosphate ratio: Increase (Human)  ^1^Carbonate to Phosphate ratio: Increase ^178^  Increased resorption (Osteoclastogenesis) | Functional implication of SNPs from literature resulting in elevated homocysteine and enhanced osteoclastogenesis (Figure 1D), Transcriptomics Pathway annotation analysis showing accumulation of homocysteine (Figure 2G), Folate (Figure 2A) and RANKL-RANK signaling (Figure 2H), Proteomics pathway annotation analysis showing the involvement of folate (Figure 3B), Pathway annotation analysis for AVNFH metabolomics showing the involvement of cysteine, methionine and Vitamin B_6_ metabolism as shown in Table 1, Pathway annotation analysis of Vitamin B_6_, B_12_ and folate co-factor dependent protein network showing the involvement of folate and vitamin B12 metabolism Figure 4C). Analysis of integration of various -Omic analyses reveals involvement of increased Osteoclastogenesis (Figure 6B).  Increased homocysteine with concomitant decrease in Vitamin B_6_ and B_12_ in AVNFH patient’s blood plasma (Metabolomics)^1^, Elevated RANKL in AVNFH patient’s blood plasma (Figure 8G). Homocysteine induces RANKL expression in osteoblasts ^32^ |
| **CT Scan Human AVNFH bone** | |
| **Parameter and its inference** | **Description of Molecular signatures correlating with the inference of the parameters** |
| Hounds field unit: Low  ^1^  Decreased Bone mineral density also indicative of decreased mineralization and increased bone degradation. | Functional implication of SNP (rs2200287) in RANKL leading to reduced bone mineral density (Figure 1D), Transcriptomics Pathway annotation analysis showing accumulation of homocysteine (Figure 2G) RANKL-RANK signaling (Figure 2H), Analysis of integration of various -Omic analyses reveals involvement of increased Osteoclastogenesis (Figure 6B).  Increased homocysteine in AVNFH patient blood plasma as part of metabolomic analysis ^1^, Elevated RANKL in AVNFH patient blood plasma (Figure 8G), Homocysteine inhibits differentiation of mesenchymal stem cells (ALP staining, Figure 8C) and mineralization by differentiated osteoblast (Alizarin red staining, Figure 8E), Analysis of integration of various -Omic analyses reveals involvement of increased Osteoclastogenesis (Figure 6C), Homocysteine has been shown to decrese tibial bone mineral density ^38^ Homocysteine induces increased RANKL expression in osteoblasts ^32^, |
| **Histopathology Human AVNFH bone** | |
| **Parameter and its inference** | **Description of Molecular signatures correlating with the inference of the parameters** |
| Increased number of Empty Lacunae  ^1^  Indicative of loss of osteocytes and death | Increased homocysteine in AVNFH patient blood plasma as part of metabolomic analysis ^1^, Elevated RANKL in AVNFH patient blood plasma (Figure 8G), Homocysteine inhibits differentiation of mesenchymal stem cells (ALP staining, Figure 8D) and mineralization by differentiated osteoblast (Alizarin red staining, Figure 8E).  Hyperhomocystenemia induces death of osteocytes ^181^ |
| Reverse cement lines  ^1^  Indicative of extensive bone remodelling (Increased osteoclast and osteoblast activity) | Functional implication of SNPs from literature resulting enhanced osteoclastogenesis (Figure 1D). Analysis of integration of various -Omic analyses reveals involvement of increased Osteoclastogenesis (Figure 6B)  Elevated levels of CTX, an independent bone resorption marker in AVNFH patient blood sera ^1^, IHC showing increased osteocalcein staining ^1^ |
| **Histopathology in Rat AVNFH bone** | |
| **Parameter and its inference** | **Description of Molecular signatures correlating with the inference of the parameters** |
| Poorly vascularized fibrous marrow in necrotic area ^182^  Indicative of Hypoxia | Integration of -Omic analyses showed involvement of HIF-1 signaling in AVNFH as shown in KEGG pathways (Figure 5C), Analysis of integration of various -Omic analyses reveals involvement of Hypoxia (Figure 6C),  HIF-1 alpha (A marker of hypoxia) is increased in its expression in the fibrosis and the interface area which is being repaired, adjacent to necrotic region in AVNFH femoral heads ^183^. |
| Presence of osteoclasts in the eroded bone surfaces  Indicates increased osteoclastogenesis | Functional implication of SNPs from literature resulting in elevated homocysteine and enhanced osteoclastogenesis (Figure 1D), Transcriptomics Pathway annotation analysis showing accumulation of homocysteine (Figure 2G), Analysis of integration of various -Omic analyses reveals involvement of increased Osteoclastogenesis (Figure 6B).  Increased homocysteine in AVNFH patient blood plasma ^1^, Homocysteine induces increased RANKL expression in osteoblasts ^32^, Elevated RANKL in AVNFH patient blood plasma (Figure 8G), |
| **Histology Human AVNFH bone** | |
| **Parameter and its inference** | **Description of Molecular signatures correlating with the inference of the parameters** |
| Presence of pronouncedly higher intravascular lipid content and small vessel wall thickening in AVNFH femoral heads. ^184^  Indicative of Coagulation and Vasconstriction | Functional implication of SNPs from literature resulting enhanced vasoconstriction (Figure 1D), Pathway annotation analysis of AVNFH SNP genes showing angiogenesis and endothelin pathways (Figure 1A) and AVNFH patient bone tissue proteomics showing complement and coagulation cascades (Figure 3C), Analysis of integration of various -Omic analyses reveals involvement of increased Coagulation and Vasoconstriction (Figure 6D)  Elevated levels of blood sodium in AVNFH patients ^1^ contributing to hypercoagulability ^1561^Presence of anticardiolipin antibodies in the blood of AVNFH patients having antiphospholipid syndrome ^185^ Elevated levels of Von Willebrand factor, a clotting protein was seen in the blood plasma of AVNFH patients ^47^, Presence of 2.3 fold higher microparticles seen in AVNFH patients having sickle cell anemia ^16^. |
| **Immunohistochemistry Human AVNFH bone** | |
| **Parameter and its inference** | **Description of Molecular signatures correlating with the inference of the parameters** |
| Osteocalcein staining: High ^1^  Indicates excessive bone remodelling | Pathway annotation analysis of AVNFH SNP genes showing the involvement of BMP6 in endochondral ossification (Figure 1A), Analysis of integration of various -Omic analyses reveals involvement of increased Endochondral ossification (Figure 6A)  Increased expression of bone morphogenetic proteins (BMP2, 4, 6 and 7) in osteoclasts generated using co-culture of mouse bone marrow cells and mouse calvarial osteoblasts. ^186^ |
| **Immunohistochemistry Human AVNFH bone** | |
| **Parameter and its inference** | **Description of Molecular signatures correlating with the inference of the parameters** |
| RANK and RANKL staining: High in AVNFH  ^13^  Increased osteoclastogenesis | Transcriptomics Pathway annotation analysis showing RANKL-RANK signaling (Figure 2H), Analysis of integration of various -Omic analyses reveals involvement of increased Osteoclastogenesis (Figure 6B), Elevated RANKL in AVNFH patient blood plasma (Figure 8G) |
| ALP staining: Low  ^13^  Reduced ostoblastogenesis and function | Homocysteine inhibits differentiation of mesenchymal stem cells (ALP staining, Figure 8D) and mineralization by differentiated osteoblast (Alizarin red staining, Figure 8E). |
| TRAP staining: High  ^13^  Indicative of increased osteoclastogenesis | Functional implication of SNPs from literature resulting enhanced osteoclastogenesis (Figure 1D). Analysis of integration of various -Omic analyses reveals involvement of increased Osteoclastogenesis (Figure 6B), Elevated levels of CTX, an independent bone resorption marker in AVNFH patient blood sera ^1^ |
| RUNX2, BMP2, BMP7 and OPG staining: High in sclerotic region ^13^  Low BMP2 and high BMP7 in necrotic region  Indicative of increased mineralization | Observation of high HU value for 1/6 of AVNFH patient bone as there were lots of sclerotic areas observed in the observed AVNFH patient bone ^1^ |
| **Immunohistochemistry Human AVNFH bone** | |
| **Parameter and its inference** | **Description of Molecular signatures correlating with the inference of the parameters** |
| Factor VIII staining: Undistinguishable in AVNFH femoral heads  ^187^  Indicative of Coagulation and Vasoconstriction | Functional implication of SNPs from literature resulting enhanced vasoconstriction (Figure 1D), Pathway annotation analysis of AVNFH SNP genes showing angiogenesis and endothelin pathways (Figure 1A) and AVNFH patient bone tissue proteomics showing complement and coagulation cascades (Figure 3C), Analysis of integration of various -Omic analyses reveals involvement of increased Coagulation and Vasoconstriction (Figure 6D)  Elevated levels of blood sodium in AVNFH patients ^1^ contributing to hypercoagulability ^156^ ^1^Presence of anticardiolipin antibodies in the blood of AVNFH patients having antiphospholipid syndrome ^185^ Elevated levels of Von willebrand factor, a clotting protein was seen in the blood plasma of AVNFH patients ^47^, Presence of 2.3 fold higher microparticles seen in AVNFH patients having sickle cell anemia ^16^, |
| **MRI Pig AVNFH bone** | |
| **Parameter and its inference** | **Description of Molecular signatures correlating with the inference of the parameters** |
| Lack of epiphyseal gadolilnium enhancement in all piglet models of AVNH (after 48 hours of induction of AVNFH) 188  Indicates coagulation and vasoconstriction | Functional implication of SNPs from literature resulting enhanced vasoconstriction (Figure 1D). Pathway annotation analysis of AVNFH SNP genes showing angiogenesis and endothelin pathways (Figure 1A ) and AVNFH patient bone tissue proteomics showing complement and coagulation cascades (Figure 3C), Analysis of integration of various -Omic analyses reveals involvement of increased coagulation and vasoconstriction(Figure 6D)  Presence of anticardiolipin antibodies in the blood of AVNFH patients having antiphospholipid syndrome ^185^, Elevated levels of Von willebrand factor, a clotting protein was seen in the blood plasma of AVNFH patients ^47^, Presence of 2.3 fold higher microparticles seen in AVNFH patients having sickle cell anemia ^16^ |
| **BOLD MRI Human AVNFH bone** | |
| **Parameter and its inference** | **Description of Molecular signatures correlating with the inference of the parameters** |
| R2*mapping:  Significantly low in AVNFH femoral heads  ^50^  Indicates coagulation and vasoconstriction | Functional implication of SNPs from literature resulting enhanced vasoconstriction (Figure 1D). Pathway annotation analysis of AVNFH SNP genes showing angiogenesis and endothelin pathways (Figure 1A ) and AVNFH patient bone tissue proteomics showing complement and coagulation cascades (Figure 3C), Analysis of integration of various -Omic analyses reveals involvement of increased coagulation and vasoconstriction(Figure 6D)  Elevated levels of blood sodium in AVNFH patients ^1^ contributing to hypercoagulability ^156^, Presence of anticardiolipin antibodies in the blood of AVNFH patients having antiphospholipid syndrome ^185^, Elevated levels of Von willebrand factor, a clotting protein was seen in the blood plasma of AVNFH patients ^47^, Presence of 2.3 fold higher microparticles seen in AVNFH patients having sickle cell anemia ^16^ |
| **MRI Piglet AVNFH bone** | |
| **Parameter and its inference** | **Description of Molecular signatures correlating with the inference of the parameters** |
| Low resolution T1W imaging reveals abnormally thickened cartilaginous epiphysis 189  Indicative of cessation of endochondral ossification | Pathway annotation analysis of AVNFH SNP genes showing the upregulation of BMP6, TGFB1 and RUNX2 in endochondral ossification (Figure 1A), Analysis of integration of various -Omic analyses reveals involvement of endochondral ossification (Figure 6A) |
| High resolution T2W mapping revealed an absence of trilaminar signal pattern in ischemic cartilagionous epiphysis  ^189^  Indicative of altered collagen fiber orientation and concentration | Pathway annotation analysis of AVNFH SNP genes showing activation of matrix metalloproteinases (Figure 1C), Increased expression of MMP2 and MMP7 in necrotic region of AVNFH femoral heads leading to destruction of collagen matrix ^190^  Presence of disrupted lamellar plates containing disorganized collagen bundles ^1^. Homocysteine inhibits the process of collagen cross linking by inhibiting the enzyme lysyl oxidase (LOX1) ^59^ |
| **MRI Human AVNFH bone** | |
| **Parameter and its inference** | **Description of Molecular signatures correlating with the inference of the parameters** |
| Bone resorption in AVNFH seen as early as in Stage 1 and also in later stages of AVNFH through MRI ^191^, ^192^  Indicative of Osteoclastogenesis | Functional implication of SNPs from literature resulting in elevated homocysteine and enhanced osteoclastogenesis (Figure 1D), Transcriptomics Pathway annotation analysis showing accumulation of homocysteine (Figure 2G), Folate (Figure 2A) and RANKL-RANK signaling (Figure 2H), Proteomics pathway annotation analysis showing the involvement of folate (Figure 3B), Pathway annotation analysis for AVNFH metabolomics showing the involvement of cysteine, methionine and Vitamin B_6_ metabolism as shown in Table 1, Pathway annotation analysis of Vitamin B_6_, B_12_ and folate co-factor dependent protein network showing the involvement of folate and vitamin B12 metabolism Figure 4C). Analysis of integration of various -Omic analyses reveals involvement of increased Osteoclastogenesis (Figure 6B).  Increased homocysteine with concomitant decrease in Vitamin B_6_ and B_12_ in AVNFH patient’s blood plasma (Metabolomics)^1^, Elevated RANKL in AVNFH patient’s blood plasma (Figure 8G). Homocysteine induces RANKL expression in osteoblasts ^32^, |

**Supplementary Table S7.6**

| **Sl No. as in Figure** | **Enriched Disease Term** | **AVNFH Comorbidities from literature** | **Reference** |
| --- | --- | --- | --- |
| 1 | Microangiopathy | Thrombotic microangiopathy associated with primary antiphospholipid syndrome | ^193^ |
| 2 | Bone necrosis | Necrosis of bone associated with AVNFH | ^1^ |
| 3 | Aneurysm | metaphyseal aneurysmal bone cyst | ^194^ |
| 4 | Pulmonary Embolism | pulmonary diseases | ^195^ |
| 5 | Diabetic Angiopathies | Diabetes | ^196^ |
| 6 | Thrombophilia | Heritable Thrombophilia-Hypofibrinolysis | ^197^ |
| 7 | Hyperthyroidism | Hyperthyroidism | ^198^ |
| 8 | Cerebrovascular events | Cardiovascular and Cerebrovascular events associated with AVNFH | ^199^ |
| 9 | Anemia, Sickle cell | sickle cell disease | ^200^ |
| 10 | Eclampsia | Osteonecrosis associated with pregnancy | ^201^ |
| 11 | Nephrotic syndrome and Kidney failure | AVNFH in chronic renal disease | ^202^ |
| 12 | Homocysteine and Pyridoxine | Elevated levels of Homocysteine and Low levels of Vitamin B6 | ^1^ |
| 13 | Hemochromatosis | Hemochromatosis | ^203^ |

**Supplementary Table S7.7**

| **RANKL Induced Osteoclastogenesis** | **RANKL** | | | | | |
| --- | --- | --- | --- | --- | --- | --- |
| **Experiment** | Osteoclastogenesis (Vit B_6_) | | Osteoclastogenesis (Vit B_12_) | | Osteoclastogenesis (Homocysteine) | |
| **Experimental Groups** | Control | B_6_ Depletion | Control | B_12_ Depletion | Control | Homocysteine |
| **Treatment** | Conventional culture Medium | Vitamin B_6_ -free medium preconditioned with low Vit B_6_ | Conventional Culture Medium | Vitamin B_12_ -free medium preconditioned with low Vit B_12_ | Conventional Culture Medium | Medium with increasing concentrations of Homocysteine |
| **Observations** |  | Increased Osteoclast Activity |  | Increased Osteoclast Activity |  | Increased Osteoclast Activity |
| **References** | ^52^ | | ^52^ | | ^52^ | |

| **Experiment** | **CBS Deficiency Mouse Model** | **CBS Gene Knockdown** | **Osteoclastogenesis**  **(ROS)** | **Osteoclastogenesis**  **(N-acetylcysteine)** | **Osteoclastogenesis**  **(Glutathione)** |
| --- | --- | --- | --- | --- | --- |
| **Experimental Groups** | Mouse models of Cystathionine beta synthase deficiency | siRNA knockdown of CBS | Reactive Oxygen Species (ROS) | N - acetylcysteine | Glutathione |
| **Condition** | Extreme and severe homocysteinemia models |  |  | Supplementation of N-acetylcysteine antioxidant | Medium with increasing concentrations of Glutathione |
| **Observations** | Hyperhomocysteinemia and Osteoporosis phenotype. | Elevated levels of Reactive Oxygen Species (ROS) | Enhance osteoclast mediated bone resorption | Decreases osteoclast differentiation and increases bone mass | GSH attenuates RANKL-induced Bone resorption and inhibits NF-κB signaling by scavenging intracellular ROS |
| **References** | ^204^ | ^205^ | ^206^ | ^207^ | ^68^ |

| **Experiment** | **Mouse model for MUT** | **Mouse model for MUT** | **Osteoclastogenesis**  **(Vitamin B_12_)** |
| --- | --- | --- | --- |
| **Experimental Groups** | Mouse model for MUT associated Methylmalonic aciduria | Mouse model for MUT associated Methylmalonic aciduria | Vitamin B_12_ Deficiency |
| **Observations** | Exhibit hematological abnormalities such as mild macrocytic anemia, as red blood cell counts, hemoglobin and hematocrit were decreased while mean cell volume and cellular haemoglobin content were increased in mutant mice. | Exhibit low bone mineral density | Stimulates Osteoclastogenesis via Increased Homocysteine and Methylmalonic Acid |
| **References** | ^69^ | ^69^ | ^162^ |

**Legends for Supplementary figures and tables:**

**Supplementary figure S7.1:** (A) List of pathways used for subnetwork analysis and their respective subnetworks. (B) Metabolism of amino acids and derivatives network and its subnetworks location (C) Degradation of cysteine and homocysteine, (D) Choline catabolism (Pathway locations from <https://reactome.org/>)

**Supplementary figure S7.2:** (A) Metabolism of water soluble vitamins and cofactors and its subnetworks location (B) metabolism of folate and pterines, (C) Vitamin B6 activation (D) Cobalamin metabolism (Pathway locations from <https://reactome.org/>)

**Supplementary figure S7.3:** (A) Phase II conjugation network and its subnetwork location(B) Methylation (Pathway locations from <https://reactome.org/>)

**Supplementary Table S7.4:** References legend for figure 7

**Supplementary Table S7.5**: Observations from different imaging techniques and biophysical characterisation techniques correlating with the molecular signatures from the present study.

**Supplementary Table S7.6:** Reference table for figures 8 A and B for literature validation showing that the Disease terms enriched are risk factors of AVNFH.

**Supplementary Table S7.7:** Literature mining to see if knock down, knock out, co-factor depletion or supplementing downstream products of CBS could modulate RANKL induced osteoclastogenesis.

**Supplementary References:**

85. Roshandel, D. *et al.* Influence of polymorphisms in the RANKL/RANK/OPG signaling pathway on volumetric bone mineral density and bone geometry at the forearm in men. *Calcif. Tissue Int.* **89,** 446–455 (2011).

86. Boyce, B. F. & Xing, L. Functions of RANKL/RANK/OPG in bone modeling and remodeling. *Arch. Biochem. Biophys.* **473,** 139–146 (2008).

87. Cheng, B.-H. *et al.* Association between single nucleotide polymorphisms of the estrogen receptor 1 and receptor activator of nuclear factor kappa B ligand genes and bone mineral density in postmenopausal Taiwanese. *Taiwan. J. Obstet. Gynecol.* **52,** 197–203 (2013).

88. Kim, T.-H. *et al.* Genetic association study of polymorphisms in the catalase gene with the risk of osteonecrosis of the femoral head in the Korean population. *Osteoarthr. Cartil.* **16,** 1060–1066 (2008).

89. Mansuri, M. S. *et al.* Catalase (CAT) promoter and 5’-UTR genetic variants lead to its altered expression and activity in vitiligo. *Br J Dermatol* **177,** 1590–1600 (2017).

90. Callaway, D. A. & Jiang, J. X. Reactive oxygen species and oxidative stress in osteoclastogenesis, skeletal aging and bone diseases. *J. Bone Miner. Metab.* **33,** 359–370 (2015).

91. Song, Y. *et al.* Associations of IGFBP3 gene polymorphism and gene expression with the risk of osteonecrosis of the femoral head in a Han population in Northern China. *DNA Cell Biol.* **35,** 836–844 (2016).

92. Silha, J. V *et al.* Perturbations in bone formation and resorption in insulin-like growth factor binding protein-3 transgenic mice. *J. Bone Miner. Res.* **18,** 1834–1841 (2003).

93. Piao, W. *et al.* A single-nucleotide polymorphism in transferrin is associated with soluble transferrin receptor levels in Chinese adolescents. *Asia Pac. J. Clin. Nutr.* (2016).

94. Yue, Y.-B., Liu, X. & Wei, B.-F. Attenuated serum adiponectin levels are associated with disease severity in patients with non-traumatic osteonecrosis of the femoral head. *J. Pain Res.* **10,** 2387 (2017).

95. Tu, Q. *et al.* Adiponectin inhibits osteoclastogenesis and bone resorption via APPL1-mediated suppression of Akt1. *J. Biol. Chem.* **286,** 12542–12553 (2011).

96. Hirata, T. *et al.* ApoB C7623T polymorphism predicts risk for steroid-induced osteonecrosis of the femoral head after renal transplantation. *J. Orthop. Sci.* **12,** 199–206 (2007).

97. Benn, M. *et al.* Common and rare alleles in apolipoprotein B contribute to plasma levels of low-density lipoprotein cholesterol in the general population. *J. Clin. Endocrinol. Metab.* **93,** 1038–1045 (2008).

98. Sato, T., Morita, I. & Murota, S. Involvement of cholesterol in osteoclast-like cell formation via cellular fusion. *Bone* **23,** 135–140 (1998).

99. Kim, T.-H., Bae, S.-C., Lee, S.-H., Kim, S.-Y. & Baek, S.-H. Association of complement receptor 2 gene polymorphisms with susceptibility to osteonecrosis of the femoral head in systemic lupus erythematosus. *Biomed Res. Int.* **2016,** (2016).

100. Cruickshank, M. N. *et al.* Transcriptional effects of a lupus-associated polymorphism in the 5′ untranslated region (UTR) of human complement receptor 2 (CR2/CD21). *Mol. Immunol.* **52,** 165–173 (2012).

101. Thorarinsdottir, K., Camponeschi, A., Gjertsson, I. & Mårtensson, I.-L. CD 21-/low B cells: A Snapshot of a Unique B Cell Subset in Health and Disease. *Scand. J. Immunol.* **82,** 254–261 (2015).

102. Kim, T.-H. *et al.* Polymorphisms in the Annexin gene family and the risk of osteonecrosis of the femoral head in the Korean population. *Bone* **45,** 125–131 (2009).

103. Zhang, J. *et al.* Gene-based meta-analysis of genome-wide association study data identifies independent single-nucleotide polymorphisms in ANXA6 as being associated with systemic lupus erythematosus in Asian populations. *Arthritis Rheumatol.* **67,** 2966–2977 (2015).

104. Garc’\ia-Melero, A. *et al.* Annexin A6 and late endosomal cholesterol modulate integrin recycling and cell migration. *J. Biol. Chem.* **291,** 1320–1335 (2016).

105. Gramoun, A. *et al.* Fibronectin inhibits osteoclastogenesis while enhancing osteoclast activity via nitric oxide and interleukin-1$β$-mediated signaling pathways. *J. Cell. Biochem.* **111,** 1020–1034 (2010).

106. Relling, M. V *et al.* Pharmacogenetic risk factors for osteonecrosis of the hip among children with leukemia. *J. Clin. Oncol.* **22,** 3930–3936 (2004).

107. Laing, B. B. & Ferguson, L. R. Genetic variations in Vitamin D metabolism genes and the microbiome, in the presence of adverse environmental changes, increase immune dysregulation. *Austin J Nutr Metab* **2,** 1026–1037 (2015).

108. Anderson, P. H. *et al.* Vitamin D depletion induces RANKL-mediated osteoclastogenesis and bone loss in a rodent model. *J. bone Miner. Res.* **23,** 1789–1797 (2008).

109. Zhou, Z. C., Gu, S. Z., Wu, J. & Liang, Q. W. VEGF, eNOS, and ABCB1 genetic polymorphisms may increase the risk of osteonecrosis of the femoral head. *Genet Mol Res* **14,** 13688–13698 (2015).

110. Vailati, F. B. *et al.* The C allele of- 634G/C polymorphism in the VEGFA gene is associated with increased VEGFA gene expression in human retinal tissue. *Invest. Ophthalmol. Vis. Sci.* **53,** 6411–6415 (2012).

111. Trebec-Reynolds, D. P., Voronov, I., Heersche, J. N. M. & Manolson, M. F. VEGF-A expression in osteoclasts is regulated by NF-$κ$B induction of HIF-1$α$. *J. Cell. Biochem.* **110,** 343–351 (2010).

112. Baldwin, C. *et al.* Association of klotho, bone morphogenic protein 6, and annexin A2 polymorphisms with sickle cell osteonecrosis. *Blood* **106,** 372–375 (2005).

113. MARTINS, D. A. P. Associa{ç}{ã}o de variantes gen{é}ticas no gene ANXA2 no desenvolvimento de complica{ç}{õ}es cl{’\i}nicas da anemia falciforme. (Universidade Federal de Pernambuco, 2016).

114. Hong, J. M. *et al.* Association study of hypoxia inducible factor 1$α$ (HIF1$α$) with osteonecrosis of femoral head in a Korean population. *Osteoarthr. Cartil.* **15,** 688–694 (2007).

115. Wang, X. *et al.* Single nucleotide polymorphism in the microRNA-199a binding site of HIF1A gene is associated with pancreatic ductal adenocarcinoma risk and worse clinical outcomes. *Oncotarget* **7,** 13717 (2016).

116. Knowles, H. J. Hypoxic regulation of osteoclast differentiation and bone resorption activity. *Hypoxia* **3,** 73 (2015).

117. Glueck, C. J., Freiberg, R. A., Oghene, J., Fontaine, R. N. & Wang, P. Association between the T-786C eNOS polymorphism and idiopathic osteonecrosis of the head of the femur. *JBJS* **89,** 2460–2468 (2007).

118. Nakayama, M. *et al.* T- 786→ C mutation in the 5′-flanking region of the endothelial nitric oxide synthase gene is associated with myocardial infarction, especially without coronary organic stenosis. *Am. J. Cardiol.* **86,** 628–634 (2000).

119. Tsukada, T. *et al.* Evidence of association of the ecNOS gene polymorphism with plasma NO metabolite levels in humans. *Biochem. Biophys. Res. Commun.* **245,** 190–193 (1998).

120. Sofowora, G. *et al.* In-vivo effects of Glu298Asp endothelial nitric oxide synthase polymorphism. *Pharmacogenet. Genomics* **11,** 809–814 (2001).

121. Collin-Osdoby, P., Rothe, L., Bekker, S., Anderson, F. & Osdoby, P. Decreased nitric oxide levels stimulate osteoclastogenesis and bone resorption both in vitro and in vivo on the chick chorioallantoic membrane in association with neoangiogenesis. *J. Bone Miner. Res.* **15,** 474–488 (2000).

122. Tobe, S. W. *et al.* The impact of endothelin-1 genetic analysis and job strain on ambulatory blood pressure. *J. Psychosom. Res.* **71,** 97–101 (2011).

123. Hong, J. M. *et al.* Genetic association of angiogenesis-and hypoxia-related gene polymorphisms with osteonecrosis of the femoral head. *Exp. Mol. Med.* **42,** 376–385 (2010).

124. Mart’\inez-Rodr’\iguez, N. *et al.* Single nucleotide polymorphisms of the angiotensin-converting enzyme (ACE) gene are associated with essential hypertension and increased ACE enzyme levels in Mexican individuals. *PLoS One* **8,** (2013).

125. Nolan, V. G. *et al.* Association of single nucleotide polymorphisms in klotho with priapism in sickle cell anemia. *Br. J. Haematol.* **128,** 266–272 (2005).

126. Kato, G. J., Hebbel, R. P., Steinberg, M. H. & Gladwin, M. T. Vasculopathy in sickle cell disease: Biology, pathophysiology, genetics, translational medicine, and new research directions. *Am. J. Hematol.* **84,** 618–625 (2009).

127. Ashley-Koch, A. E. *et al.* Identification of genetic polymorphisms associated with risk for pulmonary hypertension in sickle cell disease. *Blood, J. Am. Soc. Hematol.* **111,** 5721–5726 (2008).

128. Morris, C. R. *et al.* Dysregulated arginine metabolism, hemolysis-associated pulmonary hypertension, and mortality in sickle cell disease. *Jama* **294,** 81–90 (2005).

129. Björkman, A. *et al.* Factor V Leiden and prothrombin gene mutation: risk factors for osteonecrosis of the femoral head in adults. *Clin. Orthop. Relat. Res.* **425,** 168–172 (2004).

130. Ceelie, H., Spaargaren-van Riel, C. C., Bertina, R. M. & Vos, H. L. G20210A is a functional mutation in the prothrombin gene; effect on protein levels and 3′-end formation. *J. Thromb. Haemost.* **2,** 119–127 (2004).

131. de Stefano, V., Chiusolo, P., Paciaroni, K. & Leone, G. Epidemiology of factor V Leiden: clinical implications. in *Seminars in thrombosis and hemostasis* **24,** 367–379 (1998).

132. Cui, Y., Kaisaierjiang, A., Cao, P., Wu, Z.-Y. & Lv, Q. Association of apolipoprotein A5 genetic polymorphisms with steroid-induced osteonecrosis of femoral head in a Chinese Han population. *Diagn. Pathol.* **9,** 229 (2014).

133. Chen, X. *et al.* Hypercoagulation and elevation of blood triglycerides are characteristics of Kawasaki disease. *Lipids Health Dis.* **14,** 166 (2015).

134. Wang, Z. *et al.* Association of a polymorphism in PON-1 gene with steroid-induced osteonecrosis of femoral head in Chinese Han population. *Diagn. Pathol.* **8,** 186 (2013).

135. Yilmaz, N. Relationship between paraoxonase and homocysteine: crossroads of oxidative diseases. *Arch. Med. Sci. AMS* **8,** 138 (2012).

136. Genoud, V., Lauricella, A. M., Kordich, L. C. & Quintana, I. Impact of homocysteine-thiolactone on plasma fibrin networks. *J. Thromb. Thrombolysis* **38,** 540–545 (2014).

137. Buchel, O. *et al.* Nodular regenerative hyperplasia, portal vein thrombosis, and avascular hip necrosis due to hyperhomocysteinaemia. *Gut* **54,** 1021–1023 (2005).

138. Chai, W. *et al.* Genetic association between methylenetetrahydrofolate reductase gene polymorphism and risk of osteonecrosis of the femoral head. *Biomed Res. Int.* **2015,** (2015).

139. Ho, V., Massey, T. E. & King, W. D. Influence of thymidylate synthase gene polymorphisms on total plasma homocysteine concentrations. *Mol. Genet. Metab.* **101,** 18–24 (2010).

140. Chao, Y.-C., Wang, S.-J., Chu, H.-C., Chang, W.-K. & Hsieh, T.-Y. Investigation of alcohol metabolizing enzyme genes in Chinese alcoholics with avascular necrosis of hip joint, pancreatitis and cirrhosis of the liver. *Alcohol Alcohol.* **38,** 431–436 (2003).

141. Lumeng, L. & Li, T.-K. Vitamin B 6 Metabolism in Chronic Alcohol Abuse: Pyridoxal phosphate levels in plasma and the effects of acetaldehyde on pyridoxal phosphate synthesis and degradation in human erythrocytes. *J. Clin. Invest.* **53,** 693–704 (1974).

142. Halsted, C. H. *et al.* Ethanol feeding of micropigs alters methionine metabolism and increases hepatocellular apoptosis and proliferation. *Hepatology* **23,** 497–505 (1996).

143. Lu, M.-L. *et al.* Risks of factor V rs6020 or methylenetetrahydrofolate reductase rs12121543 polymorphism with hyperhomocysteinemia in the development of osteonecrosis of the femoral head. *J. Hip Surg.* **1,** 61–66 (2017).

144. Xie, X.-H., Wang, X.-L., Yang, H.-L., Zhao, D.-W. & Qin, L. Steroid-associated osteonecrosis: Epidemiology, pathophysiology, animal model, prevention, and potential treatments (an overview). *J. Orthop. Transl.* **3,** 58–70 (2015).

145. Clayer, M. & Bruckner, J. Occult hypoxia after femoral neck fracture and elective hip surgery. *Clin. Orthop. Relat. Res.* **370,** 265–271 (2000).

146. Boyde, A. & Hobdell, M. H. Scanning electron microscopy of lamellar bone. *Zeitschrift f{ü}r Zellforsch. und mikroskopische Anat.* **93,** 213–231 (1968).

147. Blaise, S. *et al.* Mild neonatal hypoxia exacerbates the effects of vitamin-deficient diet on homocysteine metabolism in rats. *Pediatr. Res.* **57,** 777–782 (2005).

148. Arnett, T. R. *et al.* Hypoxia is a major stimulator of osteoclast formation and bone resorption. *J. Cell. Physiol.* **196,** 2–8 (2003).

149. Chen, X.-Q., Dong, J., Niu, C.-Y., Fan, J.-M. & Du, J.-Z. Effects of hypoxia on glucose, insulin, glucagon, and modulation by corticotropin-releasing factor receptor type 1 in the rat. *Endocrinology* **148,** 3271–3278 (2007).

150. Nussinovitch, U. *The heart in rheumatic, autoimmune and inflammatory diseases: pathophysiology, clinical aspects and therapeutic approaches*. (Academic Press, 2017).

151. Vince, R. V, Chrismas, B., Midgley, A. W., McNaughton, L. R. & Madden, L. A. Hypoxia mediated release of endothelial microparticles and increased association of S100A12 with circulating neutrophils. *Oxid. Med. Cell. Longev.* **2,** 2–6 (2009).

152. Medici, V. *et al.* Impaired homocysteine transsulfuration is an indicator of alcoholic liver disease. *J. Hepatol.* **53,** 551–557 (2010).

153. Tyagi, T. *et al.* Altered expression of platelet proteins and calpain activity mediate hypoxia-induced prothrombotic phenotype. *Blood, J. Am. Soc. Hematol.* **123,** 1250–1260 (2014).

154. Lu, S. C. *et al.* Changes in methionine adenosyltransferase and S-adenosylmethionine homeostasis in alcoholic rat liver. *Am. J. Physiol. Liver Physiol.* **279,** G178--G185 (2000).

155. Italiano Jr, J. E., Mairuhu, A. T. A. & Flaumenhaft, R. Clinical relevance of microparticles from platelets and megakaryocytes. *Curr. Opin. Hematol.* **17,** 578 (2010).

156. Dmitrieva, N. I. & Burg, M. B. Secretion of von Willebrand factor by endothelial cells links sodium to hypercoagulability and thrombosis. *Proc. Natl. Acad. Sci.* **111,** 6485–6490 (2014).

157. Carmel, R., Lau, K.-H. W., Baylink, D. J., Saxena, S. & Singer, F. R. Cobalamin and osteoblast-specific proteins. *N. Engl. J. Med.* **319,** 70–75 (1988).

158. De Vernejoul, M. C. *et al.* in *Metals in Bone* 321–331 (Springer, 1985).

159. Holstein, J. H. *et al.* Low serum folate and vitamin B-6 are associated with an altered cancellous bone structure in humans. *Am. J. Clin. Nutr.* **90,** 1440–1445 (2009).

160. Nishikawa, K. *et al.* DNA methyltransferase 3a regulates osteoclast differentiation by coupling to an S-adenosylmethionine--producing metabolic pathway. *Nat. Med.* **21,** 281 (2015).

161. Utting, J. C. *et al.* Hypoxia inhibits the growth, differentiation and bone-forming capacity of rat osteoblasts. *Exp. Cell Res.* **312,** 1693–1702 (2006).

162. Vaes, B. L. T. *et al.* Vitamin B 12 deficiency stimulates osteoclastogenesis via increased homocysteine and methylmalonic acid. *Calcif. Tissue Int.* **84,** 413–422 (2009).

163. Yamamoto, I., Potts Jr, J. T. & Segre, G. V. Glucocorticoids increase parathyroid hormone receptors in rat osteoblastic osteosarcoma cells (ROS 17/2). *J. Bone Miner. Res.* **3,** 707–712 (1988).

164. Roman-Garcia, P. *et al.* Vitamin B 12--dependent taurine synthesis regulates growth and bone mass. *J. Clin. Invest.* **124,** 2988–3002 (2014).

165. Tsai, Y.-H. *et al.* Suppression of ornithine decarboxylase promotes osteogenic differentiation of human bone marrow-derived mesenchymal stem cells. *FEBS Lett.* **589,** 2058–2065 (2015).

166. Zhou, C. *et al.* Taurine promotes human mesenchymal stem cells to differentiate into osteoblast through the ERK pathway. *Amino Acids* **46,** 1673–1680 (2014).

167. Takedachi, M. *et al.* CD73-generated adenosine promotes osteoblast differentiation. *J. Cell. Physiol.* **227,** 2622–2631 (2012).

168. Villa, I. *et al.* Betaine promotes cell differentiation of human osteoblasts in primary culture. *J. Transl. Med.* **15,** 132 (2017).

169. Vijayan, V., Khandelwal, M., Manglani, K., Gupta, S. & Surolia, A. Methionine down-regulates TLR 4/MyD 88/NF-$κ$ B signalling in osteoclast precursors to reduce bone loss during osteoporosis. *Br. J. Pharmacol.* **171,** 107–121 (2014).

170. Teramachi, J. *et al.* Adenosine abolishes MTX-induced suppression of osteoclastogenesis and inflammatory bone destruction in adjuvant-induced arthritis. *Lab. Investig.* **91,** 719–731 (2011).

171. Tera, T. de M., Nascimento, R. D., Prado, R. F. do, Santamaria, M. P. & JARDINI, M. A. N. Immunolocalization of markers for bone formation during guided bone regeneration in osteopenic rats. *J. Appl. Oral Sci.* **22,** 541–553 (2014).

172. Herrmann, M. *et al.* Hyperhomocysteinemia induces a tissue specific accumulation of homocysteine in bone by collagen binding and adversely affects bone. *Bone* **44,** 467–475 (2009).

173. Kerins, D. M., Koury, M. J., Capdevila, A., Rana, S. & Wagner, C. Plasma S-adenosylhomocysteine is a more sensitive indicator of cardiovascular disease than plasma homocysteine. *Am. J. Clin. Nutr.* **74,** 723–729 (2001).

174. Imbard, A. *et al.* High homocysteine induces betaine depletion. *Biosci. Rep.* **35,** (2015).

175. Piek, C. J., Hazewinkel, H. A. W., Wolvekamp, W. T. C., Nap, R. C. & Mey, B. P. Long term follow-up of avascular necrosis of the femoral head in the dog. *J. Small Anim. Pract.* **37,** 12–18 (1996).

176. Lever, M., George, P. M., Dellow, W. J., Scott, R. S. & Chambers, S. T. Homocysteine, glycine betaine, and N, N-dimethylglycine in patients attending a lipid clinic. *Metabolism* **54,** 1–14 (2005).

177. Xiao, W. *et al.* Iron overload increases osteoclastogenesis and aggravates the effects of ovariectomy on bone mass. *J Endocrinol* **226,** 121–134 (2015).

178. Aruwajoye, O. O., Kim, H. K. W. & Aswath, P. B. Bone apatite composition of necrotic trabecular bone in the femoral head of immature piglets. *Calcif. Tissue Int.* **96,** 324–334 (2015).

179. Baggott, J. E. & Tamura, T. Homocysteine, iron and cardiovascular disease: a hypothesis. *Nutrients* **7,** 1108–1118 (2015).

180. Conn, P. M. *Animal models for the study of human disease*. (Academic Press, 2017).

181. Vijayan, V. & Gupta, S. in *Non-Proteinogenic Amino Acids* (IntechOpen, 2018).

182. Zidan, R. A. & Elnegris, H. M. Effect of homocysteine on the histological structure of femur in young male albino rats and the possible protective role of folic acid. *J. Histol. Histopathol.* **2,** 16 (2015).

183. Li, W. *et al.* Distribution of TRAP-positive cells and expression of HIF-1$α$, VEGF, and FGF-2 in the reparative reaction in patients with osteonecrosis of the femoral head. *J. Orthop. Res.* **27,** 694–700 (2009).

184. Cheras, P. A., Freemont, A. J. & Sikorski, J. M. Intraosseous thrombosis in ischemic necrosis of bone and osteoarthritis. *Osteoarthr. Cartil.* **1,** 219–232 (1993).

185. Tektonidou, M. G., Malagari, K., Vlachoyiannopoulos, P. G., Kelekis, D. A. & Moutsopoulos, H. M. Asymptomatic avascular necrosis in patients with primary antiphospholipid syndrome in the absence of corticosteroid use: a prospective study by magnetic resonance imaging. *Arthritis Rheum.* **48,** 732–736 (2003).

186. Garimella, R. *et al.* Expression and synthesis of bone morphogenetic proteins by osteoclasts: a possible path to anabolic bone remodeling. *J. Histochem. Cytochem.* **56,** 569–577 (2008).

187. Starklint, H., Lausten, G. S. & Arnoldi, C. C. Microvascular obstruction in avascular necrosis Immunohistochemistry of 14 femoral heads. *Acta Orthop. Scand.* **66,** 9–12 (1995).

188. Shapiro, F. *et al.* Femoral head deformation and repair following induction of ischemic necrosis: a histologic and magnetic resonance imaging study in the piglet. *J. Bone Jt. Surgery. Am. Vol.* **91,** 2903 (2009).

189. Babyn, P. S. *et al.* MRI of the cartilaginous epiphysis of the femoral head in the piglet hip after ischemic damage. *J. Magn. Reson. Imaging* **8,** 717–723 (1998).

190. Yamasaki, K. *et al.* Angiogenic microRNA-210 is present in cells surrounding osteonecrosis. *J. Orthop. Res.* **30,** 1263–1270 (2012).

191. Stoica, Z. *et al.* Imaging of avascular necrosis of femoral head: familiar methods and newer trends. *Curr. Heal. Sci. J.* **35,** 23 (2009).

192. Khaladkar, M. S. *et al.* Rapid magnetic resonance imaging protocol for detecting femoral head avascular necrosis: A case series-it’s utility in the general population in developing countries. *Med. J. Dr. DY Patil Univ.* **8,** 189 (2015).

193. Rueda, J. C., Duque, M. A. Q., Mantilla, R. D. & Iglesias-Gamarra, A. Osteonecrosis and antiphospholipid syndrome. *JCR J. Clin. Rheumatol.* **15,** 130–132 (2009).

194. Albiñana, J., González-Morán, G. & Morcuende, J. A. Femoral head avascular necrosis associated with metaphyseal aneurysmal bone cyst. *J. Pediatr. Orthop. B* **4,** 110–113 (1995).

195. Dima, A., Pedersen, A. B., Pedersen, L., Baicus, C. & Thomsen, R. W. Association of common comorbidities with osteonecrosis: a nationwide population-based case--control study in Denmark. *BMJ Open* **8,** (2018).

196. Lai, S.-W., Lin, C.-L. & Liao, K.-F. Real-world database examining the association between avascular necrosis of the femoral head and diabetes in Taiwan. *Diabetes Care* **42,** 39–43 (2019).

197. Glueck, C. J., Freiberg, R. A. & Wang, P. Heritable thrombophilia-hypofibrinolysis and osteonecrosis of the femoral head. *Clin. Orthop. Relat. Res.* **466,** 1034–1040 (2008).

198. Seedat, Y. K. & Randeree, M. Avascular necrosis of the hip joints in hypothyroidism. *South African Med. journal= Suid-Afrikaanse Tydskr. vir Geneeskd.* **49,** 2071–2072 (1975).

199. Sung, P.-H. *et al.* Cardiovascular and cerebrovascular events are associated with nontraumatic osteonecrosis of the femoral head. *Clin. Orthop. Relat. Res.* **476,** 865 (2018).

200. Adesina, O. O. & Neumayr, L. D. Osteonecrosis in sickle cell disease: an update on risk factors, diagnosis, and management. *Hematology* **2019,** 351–358 (2019).

201. Wood, T. J., j Hoppe, D., Winemaker, M. & Adili, A. Bilateral osteonecrosis of the femoral head during pregnancy following two corticosteroid injections: A case report and review of the literature. *Cureus* **8,** (2016).

202. Boechat, M. I., Winters, W. D., Hogg, R. J., Fine, R. N. & Watkins, S. L. Avascular necrosis of the femoral head in children with chronic renal disease. *Radiology* **218,** 411–413 (2001).

203. Rollot, F. *et al.* Hemochromatosis and femoral head aseptic osteonecrosis: a nonfortuitous association? *J. Rheumatol.* **32,** 376–378 (2005).

204. Gupta, S. *et al.* Mouse models of cystathionine $β$-synthase deficiency reveal significant threshold effects of hyperhomocysteinemia. *FASEB J.* **23,** 883–893 (2009).

205. Jia, H. *et al.* Role of the cystathionine $β$-synthase/H2S system in liver cancer cells and the inhibitory effect of quinolone-indolone conjugate QIC2 on the system. *Oncol. Rep.* **37,** 3001–3009 (2017).

206. Garrett, I. R. *et al.* Oxygen-derived free radicals stimulate osteoclastic bone resorption in rodent bone in vitro and in vivo. *J. Clin. Invest.* **85,** 632–639 (1990).

207. Cao, J. J. & Picklo, M. J. N-acetylcysteine supplementation decreases osteoclast differentiation and increases bone mass in mice fed a high-fat diet. *J. Nutr.* **144,** 289–296 (2014).

208. Kanehisa, M., Sato, Y., Furumichi, M., Morishima, K. & Tanabe, M. New approach for understanding genome variations in KEGG. Nucleic Acids Res. 47, D590--D595 (2019).

209. Kanehisa, M. Toward understanding the origin and evolution of cellular organisms. Protein Sci. 28, 1947–1951 (2019).
